# The effects of physical exercise on executive function, internalizing symptoms and externalizing symptoms in children and adolescents with attention-deficit/hyperactivity disorder: a systematic review and meta-analysis

**DOI:** 10.3389/fpsyg.2026.1984575

**Published:** 2026-10-27

**Authors:** Jinghui Shi, Xianwei Zheng, Wenzeng Wang

**Affiliations:** School of Exercise and Health, Shenyang Sport University, Shenyang, China

**Keywords:** ADHD, children and adolescents, executive function, internalizing and externalizing symptoms, meta-analysis, physical exercise

## Abstract

**Background:**

The aim of this study is to comprehensively assess the potential effects of physical exercise on executive function, internalizing symptoms and externalizing symptoms in children and adolescents with attention-deficit/hyperactivity disorder (ADHD), and to explore the moderating factors influencing these effects.

**Methods:**

We performed a systematic search of six databases (including Web of Science, PubMed, Embase, PsycNET, the Cochrane Library and SPORTDiscus). Data analysis was performed using R software, and a meta-analysis was conducted using a random-effects model to estimate the pooled effect size. In addition, subgroup analyses and meta-regression were conducted to explore potential moderating factors.

**Results:**

A total of 25 randomised controlled trials involving 1,116 participants were included. With low certainty of evidence, the pooled effect of inhibitory control was statistically significant (*g* = 0.45, *p* < 0.01). With very low certainty of evidence, the pooled effect of internalizing symptoms was statistically significant (*g* = 0.97, *p* = 0.03). The pooled effects of cognitive flexibility (*g* = 0.69, *p* = 0.054), working memory (*g* = 0.28, *p* = 0.10), and externalizing symptoms (*g* = 0.64, *p* = 0.29) did not reach statistical significance, all rated as very low certainty of evidence.

**Conclusion:**

Physical exercise may be positively associated with inhibitory control in children and adolescents with attention-deficit/hyperactivity disorder (ADHD). However, the effects of interventions show high heterogeneity, have wide confidence intervals, and exhibit limited stability.

**Systematic review registration:**

PROSPERO CRD420261380631.

## 1 Background

Attention-deficit/hyperactivity disorder (ADHD) is a highly prevalent neurodevelopmental disorder characterised by persistent and age-inappropriate symptoms of inattention, hyperactivity and impulsivity, which have a profound negative impact on the long-term developmental trajectory of children and adolescents (American Psychiatric Association, 2022; Magnus et al., 2023). In addition to these core symptoms, individuals with ADHD often exhibit severe internalizing and externalizing symptoms. Internalizing symptoms include depressiveness, anxiety and somatisation; externalizing symptoms include aggressive behavior, disciplinary problems and oppositional defiant behavior (Gillberg, 2010). Epidemiological data indicate that the global prevalence of ADHD among children and adolescents is approximately 8.0 per cent, and has been rising steadily in recent years, making it an increasingly serious global public health challenge (Ayano et al., 2023).

Executive functions are a set of higher-order cognitive processes that regulate complex goal-directed behavior (Cirino and Willcutt, 2017); It is widely accepted in academic circles that its core components include inhibitory control, working memory and cognitive flexibility (Miyake et al., 2000). A large body of research has confirmed that individuals with ADHD typically experience executive function impairments, with around 89% of them exhibiting significant deficits in at least one core component of executive function (Castellanos and Tannock, 2002; Kofler et al., 2019). These impairments not only directly undermine patients’ sustained attention, self-regulation and everyday adaptive functioning (Sadozai et al., 2024; Lee et al., 2025), but also frequently lead to a range of secondary problems, such as cognitive deficits, academic failure and social dysfunction (Bullard et al., 2024; Tamm et al., 2021). Without timely intervention, these issues will further exacerbate internalizing symptoms such as depressiveness and anxiety, as well as externalizing symptoms such as aggressive behavior, posing a long-term threat to the physical and mental well-being and social adjustment of the children affected (McKay et al., 2024; Accardo et al., 2024).

Although pharmacotherapy remains the first-line treatment for ADHD, its clinical use is limited by adverse effects such as headaches and gastrointestinal discomfort (Arnsten and Pliszka, 2011; Greydanus et al., 2019). Given the limitations of pharmacotherapy, researchers are actively exploring non-pharmacological alternative treatments; among these, physical exercise—as a safe and easily implementable intervention—has seen its potential benefits increasingly supported by research. Preliminary research suggests that a 12-week structured physical exercise intervention can enhance psychological resilience in children with ADHD and exert a positive moderating effect on internalizing and externalizing symptoms (Liang et al., 2023). Furthermore, evidence from meta-analyses indicates that physical exercise has a significant positive effect on executive function in individuals with ADHD (Yang et al., 2024; Wang et al., 2025), although findings across different sub-dimensions are not entirely consistent across studies (Liu et al., 2025a,b; Li et al., 2025).

Many existing meta-analyses have broken down executive function into inhibitory control, working memory and cognitive flexibility (Liu et al., 2025a,b; Li et al., 2025); meta-analyses of physical exercise interventions for ADHD predominantly employ traditional two-level meta-analysis models, with very few utilising three-level meta-analysis to account for non-independent effect sizes arising from multiple outcomes or intervention arms within the same original study (Liu et al., 2025a,b; Li et al., 2025). In previous meta-analyses, only a small number of studies have independently quantified internalizing symptoms (Shenning et al., 2026; Song et al., 2025), and it is rare to assess both internalizing and externalizing symptoms simultaneously within the same analytical framework. In light of these gaps in the literature, this study sets the following research objectives:

1. Previous relevant meta-analyses have all employed two-level meta-analysis models, which struggle to adequately account for multiple correlated effect sizes within the same original study, thereby potentially leading to bias in the pooled effect estimates. This study employs a three-level meta-analysis model to address this methodological shortcoming.
2. Previous meta-analyses have neither independently quantified internalizing symptoms nor simultaneously assessed internalizing and externalizing symptoms within the same analytical framework. This study fills this gap in the clinical evidence.
3. The decomposition of executive function into inhibitory control, working memory and cognitive flexibility is an analytical approach already employed in previous meta-analyses; consequently, this study treats this dimensional analysis as a secondary analysis rather than a core innovation, and further explores potential moderating factors on this basis.

## 2 Methods

This study adhered to the implementation and reporting standards set out in the 2020 PRISMA (Preferred Reporting Items for Systematic Reviews and Meta-Analyses) guidelines (Page et al., 2021); The PRISMA 2020 checklist is presented in Supplementary Table S1. Furthermore, this study has been registered with the PROSPERO under registration number CRD420261380631.

### 2.1 Data sources and search

The databases searched for this study included PubMed, Web of Science, Embase, PsycNET, SPORTDiscus and the Cochrane Library; the search period covered the period from the inception of each database to April 2026. The search strategy utilised keywords such as “children,” “adolescents,” “attention-deficit/hyperactivity disorder (ADHD),” “physical exercise” and “randomised controlled trials.” Search strategies using PubMed as an example are set out in detail in Supplementary Table S2.

### 2.2 Inclusion and exclusion criteria

Inclusion criteria (PICOS): (P) Participants were children and adolescents (aged 6 to 18 years); ADHD cases in the studies were identified through formal clinical diagnosis, validated diagnostic interviews, symptom screening scales, or a combined parent/clinician assessment. (I) The intervention group received a physical exercise intervention. (C) The control group received a non-exercise intervention. (O) Outcome measures included at least one of the following functional indicators: inhibitory control, cognitive flexibility, working memory, internalizing symptoms or externalizing symptoms. (S) The study design was a randomised controlled trial.

Exclusion criteria: (1) Studies not conducted in English; (2) Participants with comorbid mental health disorders (e.g. anxiety disorders, depression, autism spectrum disorder, post-traumatic stress disorder, etc.); (3) Literature with incomplete data or from which valid data could not be extracted; (4) Systematic reviews, research protocols and conference abstracts.

### 2.3 Literature screening and data extraction

Firstly, all articles retrieved from the databases were imported into EndNote software to eliminate duplicates. Next, two researchers independently screened the article titles and abstracts to exclude those unrelated to the research question. Finally, the full texts of potentially eligible studies were assessed to determine whether they should be included in the systematic review. Should any differences of opinion arise at any stage, the two researchers reached a consensus through consultation.

Once the literature screening was complete, two researchers extracted the data independently using Excel spreadsheets. The data extracted included: (1) basic study information (first author and year of publication); (2) participant characteristics (sample size, age, BMI), age and BMI are the original average values reported in the source text; (3) exercise parameters (type of exercise, intensity, frequency, exercise cycle, duration); (4) Mean and standard deviation of relevant outcome measures following the intervention. For studies with incomplete data, the corresponding authors will be contacted via email to request Supplementary data; studies for which complete and valid data cannot be obtained will be excluded from subsequent analysis.

### 2.4 Assessment of risk of bias and evidence grade

Two researchers independently assessed the risk of bias in all included randomised controlled trials using the Cochrane RoB 2 tool (Sterne et al., 2019). These primarily include the following dimensions: D1 Randomisation process: the study did not fully report the generation of the randomisation sequence or the allocation concealment protocol; D2 Deviation from the intended intervention: in exercise interventions, participants were unable to maintain blinding to the exercise programme; this item assesses only deviations in the implementation of the intervention; D3 Missing outcome data: Determined based on the loss-to-follow-up rate and the mechanism of data loss; D4 Outcome measurement bias: Determined by whether the outcome assessor was blinded and the type of outcome measure (objective cognitive tasks/subjective scales); D5 Selective reporting: Assessed by comparing the study registration protocol with the published report.

At the same time, the two researchers used the “Grading of Recommendations, Assessment, Development and Evaluation” (GRADE) approach to assess the quality of the evidence (Guyatt et al., 2008). In the event of any disagreement, the matter shall be resolved by an experienced third researcher, whose decision shall be final.

### 2.5 Statistical analysis

All statistical analyses and graphical representations were performed using the “metafor,” “clubSandwich” and “ggplot2” packages in R (Viechtbauer, 2010). This study employed the three-level meta-analysis model proposed by Assink and Wibbelink (Assink and Wibbelink, 2016). This model divides total variance into three levels: Level 1 (sampling), Level 2 (within studies) and Level 3 (between studies), thereby enabling the modelling of interdependence among effect sizes (Cheung, 2019). To account for situations where some studies involve multiple intervention arms (including cases with a shared control group), multiple outcome measures and multiple follow-up time points, the effect sizes derived from a single study constitute Level 2 (within-study) dependency. This model embeds the aforementioned effect sizes within their corresponding study clusters and utilises within-study random variance to uniformly account for the correlation between effect sizes of the same origin. Model parameters were estimated using restricted maximum likelihood (REML) (Rico-González et al., 2022). Cluster-robust variance estimation (CR2) was employed to correct standard errors, thereby addressing the underestimation of variance in small samples (Pustejovsky and Tipton, 2022). Concurrently, Satterthwaite’s correction for degrees of freedom was combined with a t-distribution test to control Type I errors and optimise confidence intervals.

This study selected the standardised mean difference (SMD) (Nagashima et al., 2019) as the measure of effect size. However, as most of the included studies had small sample sizes, this study uniformly adopted the bias-corrected Hedges’ g as the measure of effect size, with the following criteria: 0–0.2 indicates a negligible effect, 0.2–0.5 indicates a small effect, 0.5–0.8 indicates a moderate effect, and >0.8 indicates a large effect (Cohen, 1988). *I*^2^ is used as the primary measure of heterogeneity, with the following criteria: 0%–25% represents low heterogeneity, 25%–75% represents moderate heterogeneity, and >75% represents high heterogeneity (Nagashima et al., 2019; Nakagawa et al., 2017). Report both the 95 per cent confidence interval and the prediction interval to reflect the range of the true effect. Sensitivity analyses were conducted using leave-one-out analyses and influence diagnostics (Xu et al., 2025; Harrer et al., 2021). Studies with a Cook’s distance greater than three times the mean or an absolute studentised residual greater than 3 were considered to have an influence (Viechtbauer and Cheung, 2010; Cook and Weisberg, 1982). To investigate the sources of heterogeneity and associated factors, this study conducted regression analyses and subgroup analyses across two dimensions: participant characteristics (age, BMI) and exercise parameters (type of exercise, exercise cycle, intensity, frequency, duration) (Binney and Mansournia, 2024). For subgroups comprising three or more groups, post-hoc pairwise comparisons were performed using the Bonferroni method. In subgroup analyses, where the number of independent studies is small, the reliability of CR2 estimates is limited; the relevant results are therefore interpreted only descriptively. Publication bias was assessed using contour-enhanced funnel plots combined with the multilevel Egger test; a *p*-value < 0.05 indicates significant evidence of a small-sample effect or publication bias. (Peters et al., 2008; Egger et al., 1997).

## 3 Results

### 3.1 Literature search result

Following an initial search of various databases, a total of 3,335 articles were identified; ultimately, 25 randomised controlled trials were included in the systematic review. The various steps of this process and the corresponding screening results are shown in Figure 1.

**Figure 1:**
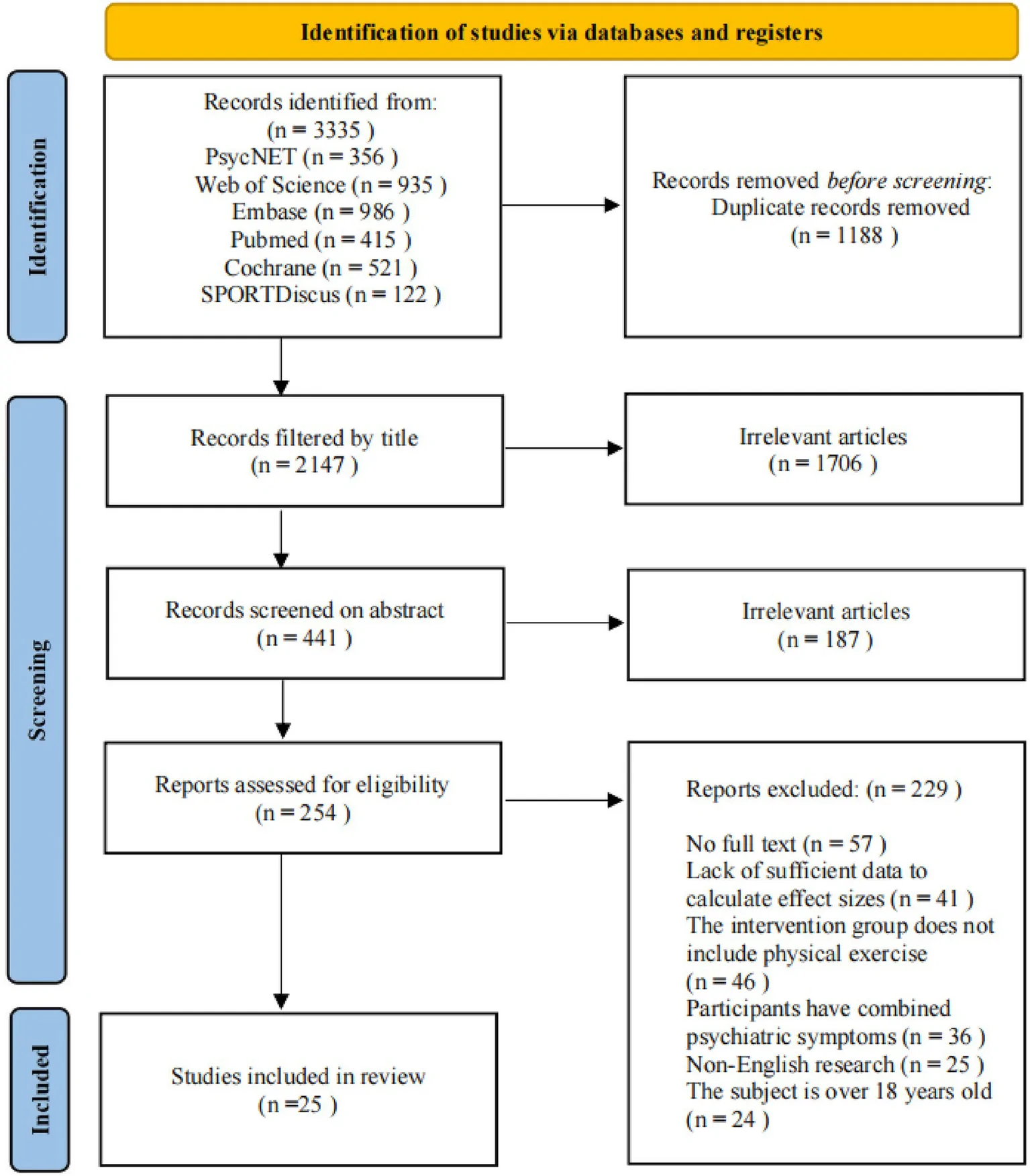
Research screening flowchart.

### 3.2 Basic information on the studies included

A total of 25 studies were included, focusing on the areas of executive function and mental disorders (see Supplementary Table S3); all studies were randomized controlled trials. In terms of participant characteristics, a total of 1,116 participants were included. The number of participants per study ranged from 12 to 94, with ages ranging from 6 to 15 years; one study (Chen, 2023) did not report age, whilst a further nine studies (Barudin-Carreiro et al., 2024; Gerber et al., 2012; Ghadamgahi Sani et al., 2022; Jovičevič et al., 2024; Chen, 2023; Oh et al., 2018; Salleg-Cabarcas et al., 2024; Smith et al., 2020; Smith et al., 2019) did not report BMI values. With regard to exercise parameters, the types of exercise involved included: aerobic exercise (Barudin-Carreiro et al., 2024; Chang et al., 2012; Silva et al., 2020; Hattabi et al., 2019; Hattabi et al., 2022; Huang et al., 2024) (6 studies); Cognitive combined exercise (Benzing et al., 2018; Benzing and Schmidt, 2019; Bustamante et al., 2016; Gerber et al., 2012; Ghadamgahi Sani et al., 2022; Hoza et al., 2015; Huang et al., 2024; Chen, 2023; Liang et al., 2022; Liu et al., 2025a,b; Smith et al., 2020; Smith et al., 2019) (12 studies) and comprehensive exercise (Huang et al., 2025; Jovičevič et al., 2024; Chen, 2023; Lee et al., 2017; Liang et al., 2024; Ludyga et al., 2022; Memarmoghaddam et al., 2016; Oh et al., 2018; Salleg-Cabarcas et al., 2024) (9 studies); six studies (Barudin-Carreiro et al., 2024; Gerber et al., 2012; Ghadamgahi Sani et al., 2022; Jovičevič et al., 2024; Smith et al., 2020; Smith et al., 2019) did not report exercise intensity; The duration of a single training session ranged from 15 to 120 minutes; a frequency of three times per week was the most common, and a 12-week intervention period was the most prevalent. We further categorized the exercise cycle into acute (a single exercise session) and chronic (exercise lasting ≥2 weeks).

### 3.3 Results of the risk of bias assessment

This study conducted an outcome-specific risk of bias assessment for the 25 included studies, with bias assessments carried out independently for each outcome measure. The overall risk of bias ratings showed that 1 study had a low risk of bias, 18 studies raised some concerns, and 6 studies had a high risk of bias (Figure 2). The primary concerns in most studies stemmed from an inadequate description of the randomization protocol; some studies exhibited potential measurement bias arising from loss to follow-up or the use of subjective scales to assess outcomes, as well as a failure to provide an open trial protocol or information on pre-registration of clinical trials.

**Figure 2:**
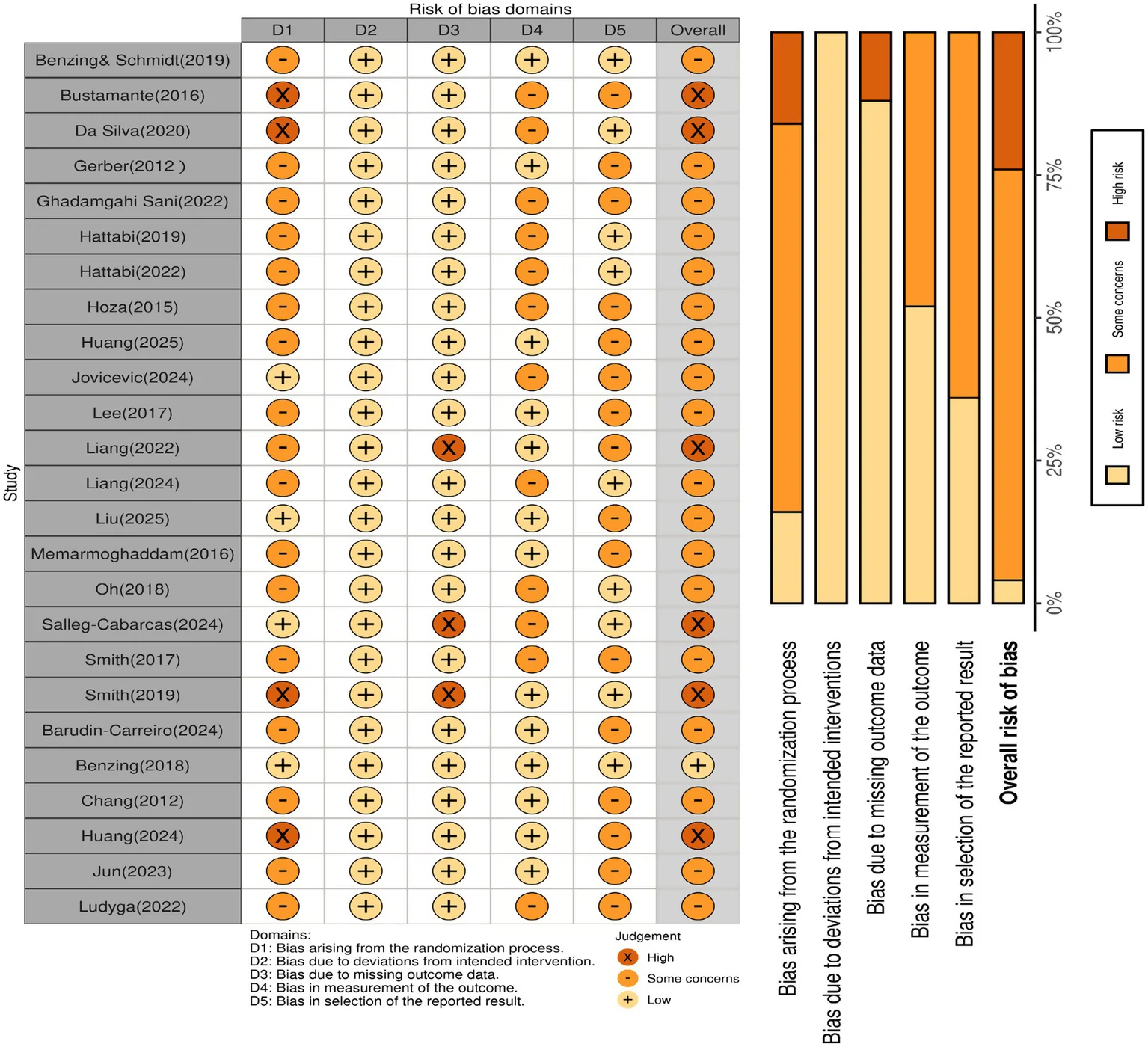
Risk of bias results.

### 3.4 Meta-analysis results

This study systematically assessed the effects of physical exercise on executive function (including inhibitory control, cognitive flexibility and working memory) and internalizing and externalizing symptoms in children and adolescents with ADHD. Meta-regression and subgroup analyses were conducted based on participant characteristics at the study level and the exercise parameters specified in each study, in order to explore potential moderating factors. Conducting multiple moderating analyses across the five outcome measures increases the risk of Type I errors; details of the relevant statistical parameters for the subgroups are provided in Supplementary Table S4. All results of the moderation analyses are exploratory in nature and are not yet sufficient to determine the optimal exercise parameters.

#### 3.4.1 Inhibitory control

A total of 16 studies (26 effect sizes) were included. The pooled effect of physical exercise on inhibitory control in children and adolescents with ADHD was statistically significant [*g* = 0.45, 95% CI [0.16, 0.74], *P* < 0.01, Q(25) = 89.17, *I*^2^-level 2 = 72%, *I*^2^-level 3 = 6%, τ^2^-level 2 = 0.30, τ^2^-level 3 = 0.02, PI (−0.76, 1.65)], (Table 1).

**Table 1:** Meta-analysis results in various fields.

Outcome	k	*n* (IC/CC)	Hedges'g (95%CI)	*P*-value	Q	*I*^**2**^ (level2)	*I*^**2**^ (level3)	τ^2^ (level2)	τ^2^ (level3)	Prediction interval
Inhibitory control	26	372/333	0.45 (0.16,0.74)	<0.01	89.17	72%	6%	0.30	0.02	(−0.76,1.65)
Cognitive flexibility	18	258/223	0.69 (−0.01,1.40)	0.054	85.24	0%	90%	0	0.88	(−1.40,2.77)
Working memory	19	300/239	0.28 (−0.06,0.62)	0.10	54.51	17%	52%	0.05	0.14	(−0.69,1.25)
Internalizing symptoms	22	189/179	0.97 (0.16,1.80)	0.03	145.28	14%	76%	0.15	0.80	(−1.17,3.11)
Externalizing symptoms	17	193/182	0.64 (−0.69,2.00)	0.29	227.34	42%	55%	1.28	1.65	(−3.17,4.45)

Hedges’g: the effect size applied in the pooled analysis; *I*^**2**^: proportion of heterogeneity; τ^2^: variance; k: number of included effect sizes; n: the number of participants in the independent study; *P*-value: statistical significance of the pooled effects; 95%CI: 95% confidence interval.

Exercise duration was statistically significant in the overall test across subgroups for the inhibitory control intervention effect (*P* = 0.03), suggesting that the effect of the exercise intervention varies across subgroups of different durations. The effect sizes for each subgroup are as follows: the short-duration subgroup (*g* = 0.58, 95% CI [0.42, 0.74], *p* < 0.01), moderate-duration subgroup (*g* = 0.13, 95% CI [−0.11, 0.36], *p* = 0.23), and long-duration subgroup (*g* = 0.83, 95% CI [0.04, 1.60], *p* = 0.04). Adjusted pairwise comparisons revealed that a statistically significant difference in intervention effect was observed only between the short-duration and moderate-duration subgroups. Further details of the subgroup analysis are provided in Supplementary Table S4.

Although no significant moderating effects were found for other exercise parameters, subgroup analyses suggested that the combined effects within the subgroups for acute exercise (*g* = 0.53, *P* < 0.01), chronic exercise (*g* = 0.44, *P* = 0.02), moderate-frequency exercise (*g* = 0.57, *P* = 0.02) and cognitive combined exercise (*g* = 0.19, *P* = 0.02) were significantly non-zero (Supplementary Table S4).

Regression analysis indicated (Supplementary Figure S1) that there was a significant association between the mean BMI at study level and the effect of exercise interventions on inhibitory control (*β* = −0.15, 95% CI [−0.27, −0.02], *P* = 0.02; 10 studies included): the higher the mean BMI in a study, the smaller the effect of the exercise intervention tended to be; No significant association was found between the mean age at study level and the intervention effect (*β* = 0.01, 95% CI [−0.13, 0.14], *P* = 0.90; 14 studies included).

#### 3.4.2 Cognitive flexibility

A total of 10 studies (18 effect sizes) were included. The pooled effect of physical exercise on cognitive flexibility in children and adolescents with ADHD did not reach statistical significance, with a P-value close to the 0.05 threshold [*g* = 0.69, 95% CI (−0.01, 1.40), *P* = 0.054, Q(17) = 85.24, *I*^2^-level 2 = 0%, *I*^2^-level 3 = 90%, τ^2^-level 2 = 0, τ^2^-level 3 = 0.88, PI (−1.40, 2.77)], (Table 1).

Subgroup analysis revealed (Supplementary Table S4) that none of the overall tests across subgroups for any of the exercise parameters were statistically significant; within each subgroup, no significant intervention effect of physical exercise on cognitive flexibility was detected.

Regression analysis indicated (Supplementary Figure S2) that the mean age at study level [*β* = 0.50, 95% CI (−0.05, 1.04), *P* = 0.07; 9 studies included] and mean BMI [*β* = 0.93, 95% CI (−0.21, 2.07), *P* = 0.09; 6 studies included] were both found to have no significant association with the intervention effect of physical exercise on improving cognitive flexibility.

#### 3.4.3 Working memory

A total of 10 studies (19 effect sizes) were included. The pooled effect of physical exercise on working memory in children and adolescents with ADHD did not reach statistical significance [*g* = 0.28, 95% CI (−0.06, 0.62), *P* = 0.10, Q(18) = 54.51, *I*^2^-level 2 = 17%, *I*^2^-level 3 = 52%, τ^2^-level 2 = 0.05, τ^2^-level 3 = 0.14, PI (−0.69, 1.25)], (Table 1).

#### 3.4.4 Internalizing symptoms

A total of 8 studies (22 effect sizes) were included. The pooled effect of physical exercise on internalizing symptoms in children and adolescents with ADHD was statistically significant [*g* = 0.97, 95% CI (0.16, 1.80), *P* = 0.03, Q(21) = 145.28, *I*^2^-level 2 = 14%, *I*^2^-level 3 = 76%, τ^2^-level 2 = 0.15, τ^2^-level 3 = 0.80, PI (−1.17, 3.11)], (Table 1).

Although no significant moderating effects were detected in the overall tests across all exercise parameters for the respective subgroups, subgroup analyses suggested that the pooled effects within the long-duration exercise (g = 1.35, *P* < 0.01) and aerobic exercise (*g* = 1.41, *P* = 0.04) subgroups were significantly non-zero (Supplementary Table S4).

Regression analysis indicated (Supplementary Figure S3) that, at the study level, mean age [*β* = 0.11, 95% CI (−0.22, 0.43), *P* = 0.45; 8 studies included] and mean BMI [*β* = 0.06, 95% CI (−0.46, 0.58), *P* = 0.73; 5 studies included] were both found to have no significant association with the intervention effect of physical exercise on internalizing symptoms.

#### 3.4.5 Externalizing symptoms

A total of 8 studies (17 effect sizes) were included. The pooled effect of physical exercise on externalizing symptoms in children and adolescents with ADHD did not reach statistical significance [*g* = 0.64, 95% CI (−0.69, 2.00), *P* = 0.29, Q(16) = 227.34, *I*^2^-level 2 = 42%, *I*^2^-level 3 = 55%, τ^2^-level 2 = 1.28, τ^2^-level 3 = 1.65, PI (−3.17, 4.45)], (Table 1).

### 3.5 Publication bias

This study employed multi-level Egger tests at Level 2 (effect size level, retaining the interdependence of multiple effect sizes within the same study) and Level 3 (study level, simultaneously estimating between-study variance and within-study effect size variance) to assess small-sample effects, and combined these with the trim-and-fill method to comprehensively evaluate publication bias; the results from the two sets of models showed some discrepancies. The Level 2 Egger test revealed a significantly asymmetric distribution of effect sizes for internalizing and externalizing symptoms (*P* < 0.05); the Level 3 Egger test indicated significant funnel asymmetry only for internalizing symptoms (*P* = 0.001). It is worth noting that funnel asymmetry merely indicates a small-sample effect and is not synonymous with publication bias; heterogeneity and sampling error can both cause asymmetry. As only eight independent studies were included for internalizing and externalizing symptoms, the statistical power of the asymmetry test was insufficient, and a negative result cannot rule out publication bias. The trim-and-fill method cannot directly handle nested effect sizes; therefore, multiple effect sizes within the same study must be merged prior to the trim-and-fill analysis. We conducted trim-and-fill analyses for both internalizing and externalizing symptoms; the results for both outcomes indicated that no missing studies needed to be included, and the pooled effects showed no significant changes after correction. However, these results are insufficient to demonstrate the robustness of the pooled effects; the trim-and-fill method has limited statistical power when the number of independent studies is small, and it cannot directly handle nested effect structures. Funnel plots for each outcome are shown in Figure 3.

**Figure 3:**
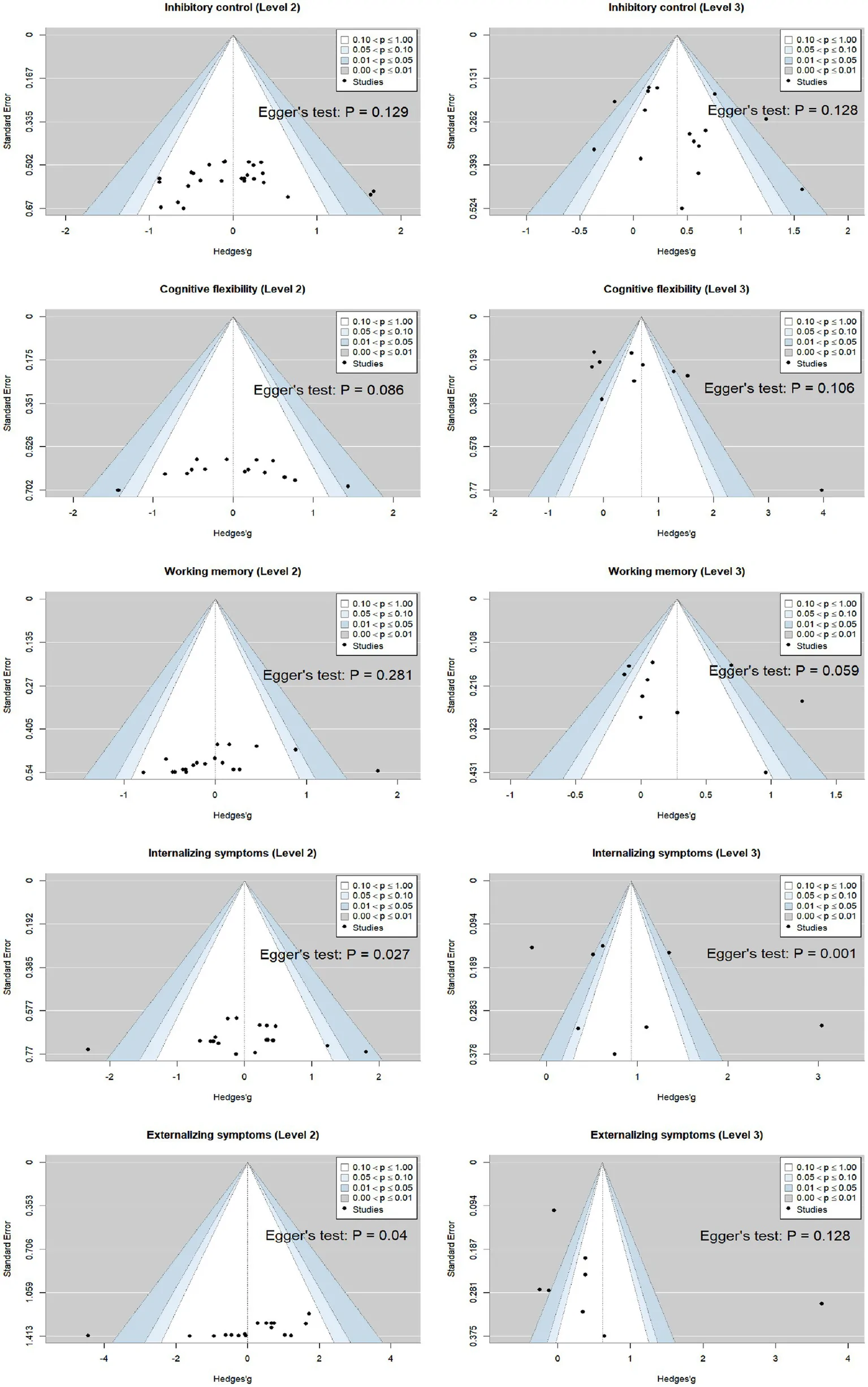
Risk of publication bias.

### 3.6 Sensitivity analysis

Results from the leave-one-out analysis indicate that the pooled effects for the indicators of inhibitory control, internalizing symptoms and externalizing symptoms remained stable after the exclusion of any single study (Supplementary Figures S4, S7 and S8). For cognitive flexibility, the pooled effect after excluding Study ID #2 (Benzing et al., 2018) was [*g* = 0.70, 95% CI (−0.01, 1.41)]; after excluding study ID#6 (Gerber et al., 2012), the pooled effect was [*g* = 0.59, 95% CI (−0.06, 1.23)]; after excluding study ID#7 (Hattabi et al., 2019), the pooled effect was [*g* = 0.63, 95% CI (−0.05, 1.30)]; the 95% confidence interval crossed the null line, rendering the pooled effect statistically insignificant (Supplementary Figure S5); For working memory, the pooled effect after excluding study ID #5 (Huang et al., 2024) was [*g* = 0.34, 95% CI (0.01, 0.67)] or, after excluding study ID #9 (Ludyga et al., 2022), the pooled effect was [*g* = 0.33, 95% CI (0.02, 0.65)]; the 95% confidence intervals did not cross the null line, and the pooled effect became statistically significant (Supplementary Figure S6). This indicates that the meta-analytic conclusions regarding cognitive flexibility and working memory are highly dependent on the aforementioned studies and exhibit poor robustness.

The influence diagnosis identified anomalous outlier effect sizes for various indicators (Supplementary Figures S9–S13): inhibitory control detected two anomalous effect sizes (ID #10 and #21); the working memory measure identified one anomalous effect size (ID #5); the internalizing symptoms measure identified one anomalous effect size (ID #21); no outliers with a significant disruptive effect were identified for the remaining measures. The aforementioned anomalous effect sizes represent important potential sources of overall heterogeneity in the corresponding measures.

### 3.7 Level of evidence

For the outcome of inhibitory control, the evidence grade was downgraded by two levels due to a high risk of bias (most included studies raised some concerns, whilst some studies had a high overall risk of bias) and serious inconsistency; it was ultimately judged to be of low quality. Low quality evidence suggests a possible trend towards improvement with exercise intervention for this outcome, but the reliability of the conclusion is limited.

For the internalizing symptoms outcome, there was a high risk of bias, serious inconsistency and serious publication bias; the evidence grade was therefore downgraded by three levels and judged to be of very low quality. For the cognitive flexibility, working memory and externalizing symptoms outcomes, there was a high risk of bias, serious inconsistency and serious imprecision; the evidence grade for each was downgraded by three levels. Among these, the risk of publication bias for cognitive flexibility and working memory was assessed as non-serious, whilst that for externalizing symptoms was assessed as unclear; none of the three were downgraded specifically due to publication bias. The quality of evidence for all three outcomes was rated as very low. Very low quality evidence indicates that existing studies are insufficient to reliably confirm the effects of exercise interventions; further validation is required through large-sample RCTs with a low risk of bias. Detailed GRADE summaries of evidence for each outcome are provided in Table 2.

**Table 2:** Evidence grade of the conclusion based on GRADE.

Outcome indicators	Sample	Assessment of evidence levels					Hedges’g (95%CI)	Level of evidence
		Risk of bias	Inconsistency	Indirectness	Imprecision	Publication bias		
Inhibitory control	705 (16RCT)	Serious	Serious	Not serious	Not serious	Not serious	0.45 (0.16,0.74)	⊕⊕○○low
Cognitive flexibility	481 (10RCT)	Serious	Serious	Not serious	Serious	Not serious	0.69 (−0.01,1.40)	⊕○○○Very low
Working memory	539 (10RCT)	Serious	Serious	Not serious	Serious	Not serious	0.28 (−0.06,0.62)	⊕○○○Very low
Internalizing symptoms	368 (8RCT)	Serious	Serious	Not serious	Not serious	Serious	0.97 (0.16,1.80)	⊕○○○Very low
Externalizing symptoms	375 (8RCT)	Serious	Serious	Not serious	Serious	Unclear	0.64 (−0.69,2.00)	⊕○○○Very low

Internalizing symptoms: publication bias was assessed as “Serious.” Level 2 and Level 3 multi-level Egger tests both indicated significant asymmetry; although the trim-and-fill analysis did not identify any missing studies, serious concerns regarding publication bias remain, and the certainty of the evidence was downgraded by one level. Externalizing symptoms: publication bias was assessed as “Unclear.” The Level 2 Egger’s test indicated asymmetry, but the Level 3 Egger’s test for the main model was not statistically significant. Due to conflicting results and a relatively small sample size, it was not possible to make a definitive judgement on publication bias; therefore, no downgrade was applied for this dimension.

## 4 Discussion

This study, based on a three-level meta-analysis of 25 randomised controlled trials, comprehensively assessed the effects of physical exercise on executive function, internalizing symptoms and externalizing symptoms in children and adolescents with ADHD, and explored the moderating roles of exercise parameters and mean baseline characteristics at the study level. Statistical results indicated that the pooled effects for inhibitory control and internalizing symptoms reached statistical significance; however, the confidence intervals for both outcomes were relatively wide and included the null value, suggesting substantial variability in intervention effects and that consistent benefits cannot be guaranteed in new populations or under different intervention conditions. No statistically significant pooled effects were detected for cognitive flexibility, working memory and externalizing symptoms; however, cognitive flexibility exhibited a marginally significant trend. Subgroup and meta-regression analyses suggest that exercise duration may moderate the intervention effect of physical exercise on inhibitory control, whilst the mean BMI at the study level is significantly and negatively associated with the effect of improvement in inhibitory control at the meta-analysis level.

### 4.1 The effects of physical exercise on executive function, internalizing symptoms and externalizing symptoms

In summary, this meta-analysis indicates that physical exercise may improve inhibitory control in some children and adolescents with ADHD; the pooled effect on internalizing symptoms is statistically significant, but the certainty of the evidence is very low, and this effect cannot yet be reliably established. This study found no significant effects of physical exercise on cognitive flexibility, working memory or externalizing symptoms. Among the five assessed outcomes, four were rated as very low certainty of evidence and only one outcome had low certainty; there is marked heterogeneity in the effects, and these effects are not consistently observed across all cognitive and symptom measures. The findings of this study are partially consistent with evidence from previous meta-analyses. This study replicated the positive findings regarding inhibitory control reported by Yang et al. (2024), Wang et al. (2025) and Liu et al. (2025a,b); the statistical effects observed for internalizing symptoms are also consistent with the conclusions of Shenning et al. (2026) and Song et al. (2025), although the certainty of the evidence for this measure in the present study is very low. However, this study failed to replicate the positive effects on cognitive flexibility and working memory reported by Yang et al. (2024), Wang et al. (2025) and Li et al. (2025); similarly, no statistically significant effects were observed for externalizing symptoms. The primary reason for the discrepancies in the conclusions of different meta-analyses may lie in the differing analytical models; most previous studies employed a traditional two-level meta-analysis model, whereas this study utilised a three-level meta-analysis to account for non-independent effect sizes arising from multiple outcomes and multiple intervention arms within the same original study. Furthermore, differences among the meta-analyses in terms of the age range of participants, exercise intervention parameters, outcome assessment tools, and inclusion and exclusion criteria for the literature may also have led to inconsistencies in the results across studies. It should be noted that the original randomised controlled trials included in the aforementioned meta-analyses reporting positive effects also carried a certain degree of risk of bias, such as inadequate randomisation and missing outcome data; these are limitations commonly found in RCTs involving exercise interventions. At the same time, the sample of original studies included in this study does not entirely overlap with that of the aforementioned meta-analyses. All of the aforementioned previous meta-analyses conducted risk of bias assessments for the included studies and utilised the GRADE system to evaluate the certainty of the evidence, applying relatively stringent evaluation criteria; consequently, the validity of their conclusions has not been fundamentally compromised. However, differences in subjective judgements regarding GRADE downgrading and the weighing of bias items across different studies have further amplified the discrepancies in conclusions and levels of evidence between the various meta-analyses.

Hypotheses proposed on the basis of previous neuroscience research suggest that the normal functioning of an individual’s executive functions relies on the coordinated activity of a neural network comprising the prefrontal cortex, hippocampus and striatum. Regular exercise may enhance blood and oxygen supply to the brain (Chen et al., 2011; Faraone and Radonjić, 2023), promote the secretion of key substances such as BDNF, dopamine and 5-hydroxytryptamine, and facilitate synaptic plasticity in the brain (Hung et al., 2018; Tsai and Pan, 2023). This may represent the potential mechanism by which exercise improves components of executive function, such as inhibitory control; however, this meta-analysis was unable to validate this biological pathway. With regard to internalizing symptoms such as anxiety and depression, exercise may suppress the overactive HPA axis, lower basal cortisol levels in the body, and mitigate the amygdala’s amplifying effect on negative emotions (Song et al., 2025; Zeibig et al., 2023), thereby regulating emotional imbalance; however, this pathway remains a theoretical hypothesis based on the literature and could not be validated by this meta-analysis.

However, this analysis did not find that physical exercise significantly improved externalizing symptoms, cognitive flexibility or working memory in this group. The fact that the measures of externalizing symptoms, cognitive flexibility and working memory were not statistically significant does not imply that the exercise intervention was ineffective; the wide confidence intervals suggest that the true effect may be either minimal or of clinical value. Externalizing symptoms are mostly manifested as hyperactivity, impulsivity, defiance and aggressive behavior (Mayes et al., 2020; Biederman et al., 2012). According to previous research, such problems are not solely caused by prefrontal cognitive deficits; they may also be influenced by a combination of factors, including parenting styles, the social environment at school, long-term behavioral habits and abnormalities in the basal ganglia dopamine pathways (Pauli-Pott and Becker, 2011; Sonuga-Barke and Halperin, 2010). Unlike measures such as inhibitory control and internalizing symptoms, physical exercise alone can only modulate the brain’s physiological state; it is difficult to alter external environmental triggers such as those in the home or school, and it is also difficult to directly correct long-standing, entrenched externalizing problem behaviors (Pauli-Pott et al., 2021; Lee et al., 2022). This hypothesis may explain why this study did not observe a significant effect of physical exercise on externalizing symptoms. The absence of a significant effect on cognitive flexibility and working memory suggests that exercise may have a selective effect on the improvement of various subcomponents of executive function, and that not all dimensions of executive function benefit equally. This result also indirectly indicates that exercise interventions have certain limitations. If the aim is to comprehensively alleviate the various symptoms of children and adolescents with ADHD, relying solely on exercise is far from sufficient; it must be combined with a range of other interventions, such as behavioral correction, family guidance and psychological counselling.

### 4.2 Moderating factor in the improvement of inhibitory control

The results of this study suggest that the effectiveness of physical exercise in improving inhibitory control appears to be influenced by the duration of the exercise.

Subgroup analyses in this study revealed that significant improvements in inhibitory control were observed in both the short-duration and long-duration exercise subgroups, whereas no significant effect was observed in the moderate-duration exercise subgroup, suggesting a possible non-linear duration effect pattern. Adjusted pairwise comparisons suggest that a statistically significant difference in intervention effects was observed only between the short-duration and moderate-duration subgroups. According to hypotheses from previous studies, short-duration exercise can rapidly increase levels of monoamine neurotransmitters such as dopamine and noradrenaline, briefly enhancing the excitability of the prefrontal cortex and exerting an acute regulatory effect on cognitive functions such as conflict inhibition (Hung et al., 2018; Tsai and Pan, 2023); long-duration exercise, on the other hand, upregulates BDNF expression through sustained neural stimulation, promoting plasticity in the prefrontal-striatal circuit and thereby yielding stable cognitive benefits (Voss et al., 2019; Kupis et al., 2021). Moderate duration falls within the transitional zone between these two effects: on the one hand, this duration may be insufficient to elicit the rapid, acute neural arousal characteristic of short-duration exercise; on the other hand, the intensity of a single session may be insufficient to drive stable neuroplastic changes (Faraone and Radonjić, 2023). Furthermore, the moderate-duration category may exhibit high intra-group heterogeneity, with included studies varying considerably in terms of exercise intensity, type of exercise and supervision methods; this may dilute the combined effects, ultimately resulting in findings that are not statistically significant. From the perspective of adherence, children and adolescents with ADHD are more likely to tolerate short-duration, low-burden programmes; interventions lasting over 60 minutes typically consist of highly structured sessions accompanied by continuous supervision; whereas programmes lasting 30–60 minutes are prone to attention lapses and reduced engagement towards the end, which may also diminish the effectiveness of the intervention (Mayes et al., 2020; Kringelbach and Deco, 2024). It should be noted that the above explanations are hypotheses based on previous literature; this meta-analysis cannot directly verify the underlying neural mechanisms.

Based on the analysis of the aforementioned moderating factors, significant improvements in inhibitory control were observed in both the short-duration and long-duration exercise subgroups. However, these findings are exploratory in nature and are not yet sufficient to inform definitive recommendations for exercise intervention programmes; further randomised controlled trials (RCTs) are required to validate this non-linear duration effect pattern.

Apart from duration, subgroup analyses revealed that the effects within the subgroups of acute exercise, chronic exercise, moderate-frequency exercise and cognitive combined exercise were significantly non-zero. At the same time, although positive effects were observed within certain strata of exercise parameters, at the overall level, exercise intensity, exercise cycle, weekly exercise frequency and exercise type did not exhibit any significant moderating effects; therefore, it cannot yet be concluded that there are statistically significant differences in intervention effects between different exercise parameters. The primary reason for this outcome is likely the small number of independent studies included in each subgroup for specific parameters, coupled with a limited total sample size, which reduced the meta-analysis’s statistical power to identify differences in effects between groups (Guyatt et al., 2011; Schulz et al., 2022). Smaller subgroup samples amplify random errors in studies, masking the actual differences in effects between different exercise parameters, and ultimately preventing the detection of moderating effects at the statistical level (Guyatt et al., 2011; Schulz et al., 2022). This also suggests that further targeted randomised controlled trials are needed in the future to accumulate evidence and further clarify the patterns of influence that exercise intensity, type, frequency and cycle have on intervention outcomes.

The results of the meta-regression analysis suggest that there is a significant negative association between the mean BMI at the study level and the intervention effect on inhibitory control: the higher the mean BMI in the included studies, the smaller the effect of physical exercise on improving inhibitory control tended to be within that study. It should be emphasised that this association is an aggregated association at the study level and cannot be directly extrapolated to individual participants. Previous studies have hypothesised that systemic inflammation associated with higher BMI levels may suppress the secretion of brain-derived neurotrophic factor (BDNF), thereby limiting exercise-induced neuroplasticity and attenuating cognitive-enhancing effects (Hanć et al., 2015; Fast et al., 2021). As the results of an exploratory analysis, this association is provided for reference in subsequent research only; it is insufficient to support the development of stratified exercise intervention programmes based on individual BMI, and this association is susceptible to confounding by other study characteristics.

### 4.3 Findings on cognitive flexibility, working memory, internalizing symptoms and externalizing symptoms

With regard to internalizing symptoms, the combined effect suggests that physical exercise may significantly alleviate internalizing symptoms in children and adolescents with ADHD. Although no statistical significance was detected in the overall tests across subgroups for any of the exercise parameters, subgroup analyses revealed that long-duration exercise and aerobic exercise had significant beneficial effects on internalizing symptoms. Meta-regression did not reveal any significant association between the mean age or mean BMI at the study level and the intervention effects on internalizing symptoms. The significant effects observed within the aforementioned subgroups merely indicate that positive effects were observed in the long-duration exercise and aerobic exercise subgroups; as the overall moderation test across subgroups was not statistically significant, it is not yet possible to infer differences in the effects of different exercise programmes. These findings are exploratory in nature and require validation by further original research.

The combined effect on cognitive flexibility did not reach statistical significance, and no significant effects were detected in any of the subgroup analyses of exercise parameters; nor were any statistically significant associations found in the meta-regression analyses for age and BMI. These findings suggest that the evidence for the benefits of exercise on cognitive flexibility remains inconsistent, with effects being susceptible to variations in study samples and intervention protocols. The pooled effect for working memory was not significant; this result suggests that the improvement in executive function resulting from exercise is dimension-selective, and that the regulatory intensity of exercise on different subsystems of the prefrontal cortex may not be consistent. There was no significant pooled effect for externalizing symptoms; in conjunction with the preceding discussion, this result suggests that exercise alone may struggle to improve externalizing behavioral problems, which are maintained by a combination of multiple environmental and neurological factors.

### 4.4 Limitations

This study has the following limitations: Firstly, there were significant differences in the exercise parameters set across studies, resulting in high heterogeneity between studies; all results of the moderation analyses are exploratory findings and are not yet sufficient to determine the optimal exercise parameters. Secondly, although a three-level meta-analysis model was used to account for multiple non-independent effects within the same study, the original RCTs had multiple methodological limitations, including inadequate reporting of randomisation, issues with outcome measurement, loss to follow-up and a lack of trial registration; consequently, the overall certainty of the evidence in this study is low. Thirdly, the total sample size is limited, with an insufficient number of studies for certain outcomes; following subgroup analysis, the sample size is further reduced, resulting in low statistical power, which may lead to the omission of potential moderating factors; therefore, the results of the stratified analyses should only be considered as exploratory references. Fourthly, the meta-regression included only mean age and mean BMI at the study level as aggregated moderating variables; such analyses carry a risk of ecological bias; the model did not account for potential confounding factors such as medication use and baseline symptom severity; consequently, the meta-regression results should be regarded as exploratory evidence only. Fifth, the methods for diagnosing ADHD varied across studies; samples based on screening or mixed reporting may not be equivalent to those with clinically diagnosed ADHD. Sixth, in almost all of the trials, the majority of participants were boys, with only a very small number of studies including a sufficient number of female participants. Consequently, the pooled evidence is primarily applicable to boys with ADHD. Finally, as only published English-language literature was included, there is a potential for publication bias.

## 5 Conclusion

Physical exercise may be positively associated with inhibitory control in children and adolescents with attention-deficit/hyperactivity disorder (ADHD). However, the effects of interventions show high heterogeneity, have wide confidence intervals, and exhibit limited stability.

## Data Availability

The original contributions presented in the study are included in the article/Supplementary material, further inquiries can be directed to the corresponding author.
