# Supplementary material for: The effects of physical exercise on executive function, internalizing symptoms and externalizing symptoms in children and adolescents with attention-deficit/hyperactivity disorder: a systematic review and meta-analysis
Source: Front Psychol. 2026 Oct 27;17:1984575. doi: 10.3389/fpsyg.2026.1984575 (PMC13747834; doi:10.3389/fpsyg.2026.1984575)
Supplement: Supplementary file 1 [file Supplementary_file_1.docx]

**Supplementary material** for

**The effects of physical exercise on executive function, internalizing symptoms and externalizing symptoms in children and adolescents with attention-deficit/hyperactivity disorder: a systematic review and meta-analysis**

**Jinghui Shi^1^, Xianwei Zheng^1^, Wenzeng Wang^1^***

**^1^ School of Exercise and Health, Shenyang Sport University, Shenyang, China**

*** Corresponding author:**

**Wenzeng Wang,**

**Email: wangwz993@163.com**

**This file includes:**

Supplementary Table

Supplementary Figure

**Supplementary Table 1** PRISMA 2020 Checklist

| **Section and Topic** | **Item #** | **Checklist item** | **Location where item is reported** |
| --- | --- | --- | --- |
| **TITLE The effects of physical exercise on executive function and mental disorders in children and adolescents with attention deficit hyperactivity disorder: a three-level meta-analysis** | | |  |
| Title | 1 | Identify the report as a systematic review. | 2 Methods |
| **ABSTRACT** | | |  |
| Abstract | 2 | See the PRISMA 2020 for Abstracts checklist. | 2 Methods |
| **INTRODUCTION** | | |  |
| Rationale | 3 | Describe the rationale for the review in the context of existing knowledge. | 1 Background |
| Objectives | 4 | Provide an explicit statement of the objective(s) or question(s) the review addresses. |  |
| **METHODS** | | |  |
| Eligibility criteria | 5 | Specify the inclusion and exclusion criteria for the review and how studies were grouped for the syntheses. | 2.2 Inclusion and exclusion criteria |
| Information sources | 6 | Specify all databases, registers, websites, organisations, reference lists and other sources searched or consulted to identify studies. Specify the date when each source was last searched or consulted. | 2.1 Data sources and search |
| Search strategy | 7 | Present the full search strategies for all databases, registers and websites, including any filters and limits used. |  |
| Selection process | 8 | Specify the methods used to decide whether a study met the inclusion criteria of the review, including how many reviewers screened each record and each report retrieved, whether they worked independently, and if applicable, details of automation tools used in the process. | 2.3 Literature screening and data  extraction |
| Data collection process | 9 | Specify the methods used to collect data from reports, including how many reviewers collected data from each report, whether they worked independently, any processes for obtaining or confirming data from study investigators, and if applicable, details of automation tools used in the process. |  |
| Data items | 10a | List and define all outcomes for which data were sought. Specify whether all results that were compatible with each outcome domain in each study were sought (e.g. for all measures, time points, analyses), and if not, the methods used to decide which results to collect. |  |
|  | 10b | List and define all other variables for which data were sought (e.g. participant and intervention characteristics, funding sources). Describe any assumptions made about any missing or unclear information. |  |
| Study risk of bias assessment | 11 | Specify the methods used to assess risk of bias in the included studies, including details of the tool(s) used, how many reviewers assessed each study and whether they worked independently, and if applicable, details of automation tools used in the process. | 2.4 Assessment of risk of bias and  evidence grade |
| Effect measures | 12 | Specify for each outcome the effect measure(s) (e.g. risk ratio, mean difference) used in the synthesis or presentation of results. | 2.5 Statistical analysis |
| Synthesis methods | 13a | Describe the processes used to decide which studies were eligible for each synthesis (e.g. tabulating the study intervention characteristics and comparing against the planned groups for each synthesis (item #5)). |  |
|  | 13b | Describe any methods required to prepare the data for presentation or synthesis, such as handling of missing summary statistics, or data conversions. |  |
|  | 13c | Describe any methods used to tabulate or visually display results of individual studies and syntheses. |  |
|  | 13d | Describe any methods used to synthesize results and provide a rationale for the choice(s). If meta-analysis was performed, describe the model(s), method(s) to identify the presence and extent of statistical heterogeneity, and software package(s) used. |  |
|  | 13e | Describe any methods used to explore possible causes of heterogeneity among study results (e.g. subgroup analysis, meta-regression). |  |
|  | 13f | Describe any sensitivity analyses conducted to assess robustness of the synthesized results. |  |
| Reporting bias assessment | 14 | Describe any methods used to assess risk of bias due to missing results in a synthesis (arising from reporting biases). | 2.4 Assessment of risk of bias and  evidence grade |
| Certainty assessment | 15 | Describe any methods used to assess certainty (or confidence) in the body of evidence for an outcome. |  |
| **RESULTS** | | |  |
| Study selection | 16a | Describe the results of the search and selection process, from the number of records identified in the search to the number of studies included in the review, ideally using a flow diagram. | 3.1 Literature search result |
|  | 16b | Cite studies that might appear to meet the inclusion criteria, but which were excluded, and explain why they were excluded. |  |
| Study characteristics | 17 | Cite each included study and present its characteristics. | 3.2 Basic information on the studies  included |
| Risk of bias in studies | 18 | Present assessments of risk of bias for each included study. | 3.3 Results of the risk of bias assessment |
| Results of individual studies | 19 | For all outcomes, present, for each study: (a) summary statistics for each group (where appropriate) and (b) an effect estimate and its precision (e.g. confidence/credible interval), ideally using structured tables or plots. | TABLE 1 Meta-analysis results in various fields |
| Results of syntheses | 20a | For each synthesis, briefly summarise the characteristics and risk of bias among contributing studies. | 3.3 Results of the risk of bias assessment |
|  | 20b | Present results of all statistical syntheses conducted. If meta-analysis was done, present for each the summary estimate and its precision (e.g. confidence/credible interval) and measures of statistical heterogeneity. If comparing groups, describe the direction of the effect. | 3.4 Meta-analysis results |
|  | 20c | Present results of all investigations of possible causes of heterogeneity among study results. |  |
|  | 20d | Present results of all sensitivity analyses conducted to assess the robustness of the synthesized results. | 3.6 Sensitivity analysis |
| Reporting biases | 21 | Present assessments of risk of bias due to missing results (arising from reporting biases) for each synthesis assessed. | 3.5 Publication bias |
| Certainty of evidence | 22 | Present assessments of certainty (or confidence) in the body of evidence for each outcome assessed. | 3.7 Level of evidence |
| **DISCUSSION** | | |  |
| Discussion | 23a | Provide a general interpretation of the results in the context of other evidence. | 4 Discussion |
|  | 23b | Discuss any limitations of the evidence included in the review. | 4.4 Limitations |
|  | 23c | Discuss any limitations of the review processes used. |  |
|  | 23d | Discuss implications of the results for practice, policy, and future research. | na |
| **OTHER INFORMATION** | | |  |
| Registration and protocol | 24a | Provide registration information for the review, including register name and registration number, or state that the review was not registered. | 2 Methods |
|  | 24b | Indicate where the review protocol can be accessed, or state that a protocol was not prepared. | na |
|  | 24c | Describe and explain any amendments to information provided at registration or in the protocol. | na |
| Support | 25 | Describe sources of financial or non-financial support for the review, and the role of the funders or sponsors in the review. | Funding |
| Competing interests | 26 | Declare any competing interests of review authors. | Conflict of interest |
| Availability of data, code and other materials | 27 | Report which of the following are publicly available and where they can be found: template data collection forms; data extracted from included studies; data used for all analyses; analytic code; any other materials used in the review. | Data availability statement |

**Supplementary Table 2** Search strategy for PubMed

| **Step** | **Search strategy** |
| --- | --- |
| #1 | (Exercise) or (Exercises) or (Exercise, Physical) or (Exercises, Physical) or (Physical Exercise) or (Physical Exercises) or (Exercise, Isometric) or (Exercises, Isometric) or (Isometric Exercises) or (Isometric Exercise) or (Exercise, Aerobic) or (Aerobic Exercise) or (Aerobic Exercises) or (Exercises, Aerobic) or (Exercise Training) or (Exercise Trainings) or (Training, Exercise) or (Trainings, Exercise) or (Physical Activity) or (Activities, Physical) or (Activity, Physical) or (Physical Activities) or (Active Breaks) or (Activity Breaks) or (Acute Exercise) or (Acute Exercises) or (Exercise, Acute) or (Exercises, Acute) |
| #2 | (Child) or (Children) or (Adolescent) or (Adolescents) or (Adolescence) or (Adolescents, Female) or (Adolescent, Female) or (Female Adolescent) or (Female AdolescentsAdolescents, Male) or (Adolescent, Male) or (Male Adolescent) or (Male Adolescents) or (Youth) or (Youths) or (Teens) or (Teen) or (Teenagers) or (Teenager) |
| #3 | (Attention Deficit Disorder with Hyperactivity) or (ADHD) or (ADDH) or (Attention Deficit Disorders with Hyperactivity) or (Attention Deficit Hyperactivity Disorders) or (Attention Deficit Hyperactivity Disorder) or (Attention Deficit-Hyperactivity Disorder) or (Attention Deficit-Hyperactivity Disorders) or (Deficit-Hyperactivity Disorder, Attention) or (Deficit-Hyperactivity Disorders, Attention) or (Disorder, Attention Deficit-Hyperactivity) or (Disorders, Attention Deficit-Hyperactivity) or (Hyperkinetic Syndrome) or (Syndromes, Hyperkinetic) or (Attention Deficit Disorder) or (Attention Deficit Disorders) or (Deficit Disorder, Attention) or (Deficit Disorders, Attention) or (Disorder, Attention Deficit) or (Disorders, Attention Deficit) or (Brain Dysfunction, Minimal) or (Dysfunction, Minimal Brain) or (Minimal Brain Dysfunction) |
| #4 | (Randomized controlled trial) or (Clinical Trials, Randomized) or (Trials, Randomized Clinical) or (Controlled Clinical Trials, Randomized) or (Randomized Clinical Trial) |
| #5 | #1 AND #2 AND #3 AND #4 |

**Supplementary Table 3** Basic features of the included studies

| First author(year) | Participant description | | | Intervention measures | | | | | | control measures | Diagnostic methods | Outcome |
| --- | --- | --- | --- | --- | --- | --- | --- | --- | --- | --- | --- | --- |
|  | Sample size (IC/CC) | Age | BMI (kg/m^2^) | Content | Type | Intensity | Duration (min) | frequency | Weeks |  |  |  |
| Barudin- Carreiro(2024) | 7/8 | 9.67 | na | Walking | AE | na | 20 | 1 | 1 | Sitting | Parent report/ doctor diagnosis | ①② |
| Benzing(2018) | 24/22 | 10.48 | 18.46 | Motion-sensing game | CME | MH | 15 | 1 | 1 | Watch video | ICD-10 | ①②③ |
| Benzing and  Schmidt(2019) | 28/23 | 10.43 | 17.87 | Motion-sensing game | CME | MH | 30 | 3 | 8 | Daily activities | ICD-10 | ①②③ |
| Bustamante(2016) | 18/16 | 9.08 | 22.15 | Structured sports games | CME | M | 60 | 5 | 10 | Sedentary games | DSM-IV | ①③⑤ |
| Chang(2012) | 20/20 | 10.43 | 16.79 | Running | AE | M | 30 | 1 | 1 | Watch video | DSM-IV | ①② |
| Da Silva(2020) | 10/10 | 12 | 19.72 | Swimming | AE | M | 45 | 2 | 8 | Daily activities | DSM-IV | ②④ |
| Gerber(2012) | 18/19 | 11.27 | na | Summer camp training | CME | na | 60 | 5 | 2.5 | Parental guidance | DSM-IV | ①② |
| Ghadamgahi  Sani(2022) | 20/20 | 7.64 | na | Perceptual motor exercises | CME | na | 42.5 | 3 | 7 | Neurofeedback | DSM–5 | ① |
| Hattabi(2019) | 20/20 | 9.85 | 17.55 | Swimming | AE | M | 90 | 3 | 12 | Daily activities | DSM-IV | ①②③ |
| Hattabi(2022) | 20/20 | 9.85 | 17.55 | Swimming | AE | M | 90 | 3 | 12 | Daily activities | DSM-IV | ④⑤ |
| Hoza(2015) | 49/45 | 6.83 | 17.29 | Structured PE class | CME | MH | 31 | 7 | 12 | Sitting | K-SADS-PL | ④⑤ |
| Huang(2024)a | 22/17 | 9.64 | 18.58 | Skipping rope | AE | MH | 60 | 2 | 8 | Daily activities | DSM-5 | ③ |
| Huang(2024)b | 16/17 | 9.15 | 18.03 | Structured PE class | CME | MH | 60 | 2 | 8 | Daily activities | DSM-5 | ③ |
| Huang(2025) | 12/12 | 10.21 | 18.04 | Roller skating | CE | MH | 80 | 2 | 24 | Daily activities | DSM-IV | ①③ |
| Jovicevic(2024) | 20/19 | 10 | na | Systematic training | CE | na | 60 | 2 | 12 | Mindfulness | DSM-5 | ④⑤ |
| Jun(2023)a | 26/24 | na | na | Football | CE | MH | 40 | 4 | 6 | Daily activities | SNAP-IV | ①②③ |
| Jun(2023)b | 24/24 | na | na | Structured PE class | CME | MH | 40 | 4 | 6 | Daily activities | SNAP-IV | ①②③ |
| Lee(2017) | 6/6 | 8.83 | 15.94 | Comprehensive exercise | CE | M | 60 | 3 | 12 | Daily activities | Parent report/ doctor diagnosis | ① |
| Liang(2022) | 39/39 | 8.33 | 16.68 | Aerobic combined with  cognitive training | CME | MH | 60 | 3 | 12 | Daily activities | K-SADS-PL | ①②③ |
| Liang(2024) | 15/15 | 8.62 | 16.3 | Various ball sports | CE | MH | 60 | 3 | 12 | Daily activities | DSM-5 | ④⑤ |
| Liu(2025) | 36/33 | 14.74 | 20.91 | Structured PE class | CME | MH | 60 | 1 | 12 | Daily activities | Parent report/ doctor diagnosis | ①④⑤ |
| Ludyga(2022) | 29/28 | 10.39 | 18.25 | Taekwondo | CE | MH | 60 | 2 | 12 | Daily activities | DSM-5 | ③ |
| Memarmoghaddam (2016) | 19/17 | 8.3 | 16.72 | Running and various  ball sports | CE | MH | 90 | 3 | 8 | Daily activities | SNAP-IV | ① |
| Oh(2018) | 17/15 | 8.15 | na | Equestrianism | CE | M | 60 | 2 | 12 | Medication therapy | DSM-IV | ④⑤ |
| Salleg- Cabarcas(2024) | 22/22 | 10.5 | na | Fighting sports game | CE | MH | 45 | 3 | 12 | Daily activities | Parent report/ doctor diagnosis | ④⑤ |
| Smith(2017) | 42/38 | 7.41 | na | Cognition, physical activity, behavior management | CME | na | 120 | 3 | 15 | Routine treatment | DSM-IV | ①②③ |
| Smith(2019) | 13/16 | 7.14 | na | Cognition, physical activity, behavior management | CME | na | 120 | 3 | 15 | Routine treatment | K-SADS-PL | ① |
| Note: ① Inhibitory control; ② Cognitive flexibility; ③ Working memory; ④ Internalizing symptoms; ⑤ Externalizing symptoms; AE: Aerobic exercise; CE: Comprehensive exercise; CME: Cognitive combined exercise; M: Moderate intensity; MH: Moderate to high intensity exercise; na indicates that there is no data for this item. | | | | | | | | | | | | |
|  |  |  |  |  |  |  |  |  |  |  |  |  |
|  |  |  |  |  |  |  |  |  |  |  |  |  |

**Supplementary Table 4** Results of subgroup analysis

| **Subgroup** | | **Outcome** | **k(n)** | **Hedges’g** | **95%CI** | **Pd** | **Q** | **I²-l2(%)** | **I²-l3** | **τ²-l2** | **τ²-l3** | **Pm** |
| --- | --- | --- | --- | --- | --- | --- | --- | --- | --- | --- | --- | --- |
| Exercise intensity | Moderate | IC | 6(126) | 0.72 | -0.59,2.00 | 0.17 | 27.14 | 82 | 0 | 0.85 | 0.00 | 0.64 |
|  | Moderate to high |  | 13(378) | 0.47 | -0.05,0.98 | 0.07 | 47.80 | 71 | 10 | 0.31 | 0.04 |  |
|  | Moderate | CF | 5(100) | 1.58 | -3.50,6.70 | 0.31 | 39.95 | 0 | 97 | 0.00 | 3.97 | 0.41 |
|  | Moderate to high |  | 7(249) | 0.42 | -0.15,0.99 | 0.10 | 9.21 | 0 | 51 | 0.00 | 0.08 |  |
|  | Moderate | IS | 13(92) | 1.06 | -0.35,2.50 | 0.08 | 44.37 | 55 | 23 | 0.37 | 0.15 | 1.00 |
|  | Moderate to high |  | 8(237) | 1.05 | -1.10,3.2 | 0.23 | 90.04 | 0 | 96 | 0.00 | 1.81 |  |
| Exercise cycle | Acute | IC | 3(101) | 0.53 | 0.42,0.64 | <0.01 | 0.03 | 0 | 0 | 0.00 | 0.00 | 0.65 |
|  | Chronic |  | 23(604) | 0.44 | 0.10,0.79 | 0.02 | 88.22 | 74 | 7 | 0.36 | 0.03 |  |
|  | Acute | CF | 6(101) | 0.18 | -1.20,1.50 | 0.63 | 10.15 | 0 | 62 | 0.00 | 0.21 | 0.22 |
|  | Chronic |  | 12(380) | 0.96 | -0.14,2.00 | 0.08 | 73.12 | 0 | 94 | 0.00 | 1.31 |  |
|  | Acute | IS | na | na | na | na | na | na | na | na | na | na |
|  | Chronic |  | 22(368) | 0.97 | 0.16,1.80 | 0.03 | 145.28 | 14 | 76 | 0.15 | 0.80 |  |
| Exercise duration | Short(≤30 min) | IC | 4(152) | **0.58** | 0.42,0.74 | <0.01 | 0.20 | 0 | 0 | 0.00 | 0.00 | 0.03 |
|  | Moderate(31-60 min) |  | 12(344) | **0.13** | -0.11,0.36 | 0.23 | 18.78 | 42 | 0 | 0.06 | 0.00 |  |
|  | Long(＞60 min) |  | 10(209) | 0.83 | 0.04,1.60 | 0.04 | 56.12 | 87 | 0 | 0.73 | 0.00 |  |
|  | Short(≤30 min) | CF | 7(152) | 0.27 | -0.50,1.00 | 0.34 | 11.23 | 0 | 56 | 0.00 | 0.15 | 0.61 |
|  | Moderate(31-60 min) |  | 7(209) | 1.37 | -1.20,4.00 | 0.19 | 43.27 | 0 | 96 | 0.00 | 2.56 |  |
|  | Long(＞60 min) |  | 4(120) | 0.54 | -8.70,9.80 | 0.59 | 26.04 | 0 | 93 | 0.00 | 1.01 |  |
|  | Short(≤30 min) | IS | 0 | na | na | na | na | na | na | na | na | 0.30 |
|  | Moderate(31-60 min) |  | 17(328) | 0.94 | -0.05,1.90 | 0.06 | 123.63 | 32 | 60 | 0.45 | 0.83 |  |
|  | Long(＞60 min) |  | 5(40) | 1.35 | 0.92,1.79 | <0.01 | 0.17 | 0 | 0 | 0.00 | 0.00 |  |
| Exercise frequency | Low(<3times) | IC | 6(170) | 0.54 | -0.14,1.20 | 0.09 | 11.52 | 25 | 35 | 0.06 | 0.09 | 0.28 |
|  | Moderate(3 times) |  | 16(390) | 0.57 | 0.12,1.00 | 0.02 | 65.02 | 82 | 0 | 0.45 | 0.00 |  |
|  | High(>3 times) |  | 4(145) | 0.00 | -1.20,1.20 | 0.99 | 5.09 | 0 | 59 | 0.00 | 0.14 |  |
|  | Low(<3 times) | CF | 7(121) | 1.01 | -1.80,3.90 | 0.34 | 32.96 | 0 | 96 | 0.00 | 3.07 | 0.93 |
|  | Moderate(3 times) |  | 7(249) | 0.52 | -0.44,1.50 | 0.18 | 28.37 | 0 | 82 | 0.00 | 0.31 |  |
|  | High(>3 times) |  | 4(111) | 0.72 | -0.94,11.00 | 0.53 | 23.16 | 0 | 92 | 0.00 | 1.22 |  |
|  | Low(<3 times) | IS | 12(160) | 0.71 | -0.04,1.50 | 0.06 | 34.07 | 78 | 0 | 0.42 | 0.00 | 0.07 |
|  | Moderate(3 times) |  | 8(114) | 1.72 | -1.20,4.60 | 0.13 | 31.66 | 0 | 90 | 0.00 | 1.27 |  |
|  | High(>3 times) |  | 2(94) | -0.16 | -2.02,1.70 | 0.48 | 0.20 | 0 | 0 | 0.00 | 0.00 |  |
| Types of Exercise | Aerobic exercise | IC | 4(95) | 0.99 | -0.89,2.90 | 0.14 | 16.57 | 83 | 0 | 0.73 | 0.00 | 0.14 |
|  | Comprehensive exercise |  | 8(122) | 0.77 | -0.12,1.70 | 0.07 | 33.96 | 80 | 0 | 0.61 | 0.00 |  |
|  | Cognitive combined exercise |  | 14(512) | 0.19 | 0.04,0.34 | 0.02 | 21.50 | 37 | 0 | 0.04 | 0.00 |  |
|  | Aerobic exercise | CF | 7(115) | 1.15 | -1.70,4.00 | 0.29 | 42.66 | 0 | 95 | 0.00 | 3.08 | 0.36 |
|  | Comprehensive exercise |  | 1(51) | na | na | na | na | na | na | na | na |  |
|  | Cognitive combined exercise |  | 10(276) | 0.49 | -0.16,1.10 | 0.11 | 39.97 | 0 | 82 | 0.00 | 0.33 |  |
|  | Aerobic exercise | IS | 8(60) | 1.41 | 0.33,2.50 | 0.04 | 30.41 | 84 | 0 | 0.83 | 0 | 0.34 |
|  | Comprehensive exercise |  | 9(145) | 1.16 | -0.85,3.20 | 0.16 | 58.21 | 0 | 91 | 0 | 1.49 |  |
|  | Cognitive combined exercise |  | 5(163) | 0.23 | -4.70,5.20 | 0.66 | 15.44 | 0 | 84 | 0 | 0.28 |  |
| Note: IC: Inhibitory Control, CF: Cognitive Flexibility, IS: Internalizing Symptoms, Hedges’g: the effect size applied in the pooled analysis; I²: proportion of heterogeneity; τ²: Variance; K: number of included effect sizes; P-value: statistical significance of the pooled effects; Pm= P value of the difference between subgroups; 95%CI: 95% confidence interval; Q: Cochran's Q statistic; Power: statistical power; Acute: a single exercise session; Chronic: exercise lasting ≥2 weeks; Aerobic exercise: cardiopulmonary endurance training without cognitive tasks; Comprehensive exercise: mixed physical fitness training without cognitive tasks; Cognitive combined exercise: a multi-component intervention combining exercise with cognitive/behavioural training; na = no data. | | | | | | | | | | | | |
|  |  |  |  |  |  |  |  |  |  |  |  |  |
|  |  |  |  |  |  |  |  |  |  |  |  |  |
|  |  |  |  |  |  |  |  |  |  |  |  |  |

**Supplementary Figure 1** Regression analysis result chart of Inhibitory control

**Supplementary Figure 2** Regression analysis result chart of cognitive flexibility

**Supplementary Figure 3** Regression analysis result chart of Internalizing symptoms

**Supplementary Figure 4** Leave-one-out sensitivity analysis of inhibitory control

**Supplementary Figure 5** Leave-one-out sensitivity analysis of cognitive flexibility

**Supplementary Figure 6** Leave-one-out sensitivity analysis of working memory

**Supplementary Figure 7** Leave-one-out sensitivity analysis of internalizing symptoms

**Supplementary Figure 8** Leave-one-out sensitivity analysis of externalizing symptoms

**Supplementary Figure 9** Inhibitory control influence analysis

**Supplementary Figure 10** Cognitive flexibility influence analysis

**Supplementary Figure 11** Working memory influence analysis

**Supplementary Figure 12** Internalizing symptoms influence analysis

**Supplementary Figure 13** Externalizing symptoms influence analysis

**Supplementary Table 5** Inhibitory control dataset

| study | mean1 | sd1 | n1 | mean2 | sd2 | n2 | es | se2 | aa | bb | inten | fre | type | age | bmi | duration | dura | week | wek |
| --- | --- | --- | --- | --- | --- | --- | --- | --- | --- | --- | --- | --- | --- | --- | --- | --- | --- | --- | --- |
| Barudin-Carreiro(2024) | 47.71 | 5.94 | 7.00 | 44.50 | 7.21 | 8.00 | 0.45 | 0.28 | 1.00 | 1.00 |  | 0.00 | 0.00 | 9.67 |  | 20.00 | 1.00 | 1.00 | 1.00 |
| Benzing(2018) | -842.00 | 162.00 | 24.00 | -959.00 | 266.00 | 22.00 | 0.53 | 0.09 | 2.00 | 2.00 | 1.00 | 0.00 | 2.00 | 10.48 | 18.46 | 15.00 | 1.00 | 1.00 | 1.00 |
| Benzing and Schmidt(2019) | -537.00 | 88.00 | 28.00 | -607.00 | 117.00 | 23.00 | 0.68 | 0.08 | 3.00 | 3.00 | 1.00 | 1.00 | 2.00 | 10.43 | 17.87 | 30.00 | 1.00 | 8.00 | 1.00 |
| Bustamante(2016) | -370.00 | 247.00 | 18.00 | -292.00 | 153.00 | 16.00 | -0.37 | 0.12 | 4.00 | 4.00 | 0.00 | 2.00 | 2.00 | 9.08 | 22.15 | 60.00 | 2.00 | 10.00 | 1.00 |
| Chang(2012) | -49.94 | 9.15 | 20.00 | -57.30 | 15.64 | 20.00 | 0.56 | 0.10 | 5.00 | 5.00 | 0.00 | 0.00 | 0.00 | 10.43 | 16.79 | 30.00 | 1.00 | 1.00 | 1.00 |
| Gerber(2012) | -90.95 | 29.23 | 18.00 | -218.46 | 283.01 | 19.00 | 0.61 | 0.11 | 6.00 | 6.00 |  | 2.00 | 2.00 | 11.27 |  | 60.00 | 2.00 | 2.50 | 1.00 |
| Ghadamgahi Sani(2022)a | -701.90 | 93.01 | 20.00 | -664.20 | 68.69 | 20.00 | -0.45 | 0.10 | 7.00 | 7.00 |  | 1.00 | 2.00 | 7.64 |  | 42.50 | 2.00 | 7.00 | 1.00 |
| Ghadamgahi Sani(2022)b | -2.90 | 2.29 | 20.00 | -4.45 | 2.11 | 20.00 | 0.69 | 0.11 | 8.00 | 7.00 |  | 1.00 | 2.00 | 7.64 |  | 42.50 | 2.00 | 7.00 | 1.00 |
| Hattabi(2019)a | -87.55 | 36.30 | 20.00 | -105.15 | 28.37 | 20.00 | 0.53 | 0.10 | 9.00 | 8.00 | 0.00 | 1.00 | 0.00 | 9.85 | 17.55 | 90.00 | 3.00 | 12.00 | 1.00 |
| Hattabi(2019)b | -12.65 | 2.13 | 20.00 | -18.70 | 2.72 | 20.00 | 2.43 | 0.18 | 10.00 | 8.00 | 0.00 | 1.00 | 0.00 | 9.85 | 17.55 | 90.00 | 3.00 | 12.00 | 1.00 |
| Huang(2025) | 40.33 | 8.28 | 12.00 | 26.17 | 9.06 | 12.00 | 1.58 | 0.23 | 11.00 | 9.00 | 1.00 | 0.00 | 1.00 | 10.21 | 18.04 | 80.00 | 3.00 | 24.00 | 2.00 |
| Jun(2023)a | 18.25 | 1.73 | 26.00 | 18.56 | 1.35 | 24.00 | -0.20 | 0.08 | 12.00 | 10.00 | 1.00 | 2.00 | 1.00 |  |  | 40.00 | 2.00 | 6.00 | 1.00 |
| Jun(2023)b | 18.31 | 1.78 | 24.00 | 18.56 | 1.35 | 24.00 | -0.16 | 0.08 | 13.00 | 10.00 | 1.00 | 2.00 | 2.00 |  |  | 40.00 | 2.00 | 6.00 | 1.00 |
| Lee(2017)a | 54.17 | 5.38 | 6.00 | 50.50 | 3.15 | 6.00 | 0.77 | 0.37 | 14.00 | 11.00 | 0.00 | 1.00 | 1.00 | 8.83 | 15.94 | 60.00 | 2.00 | 12.00 | 1.00 |
| Lee(2017)b | 48.00 | 5.44 | 6.00 | 43.33 | 12.24 | 6.00 | 0.46 | 0.35 | 15.00 | 11.00 | 0.00 | 1.00 | 1.00 | 8.83 | 15.94 | 60.00 | 2.00 | 12.00 | 1.00 |
| Liang(2022)a | -771.59 | 275.88 | 39.00 | -927.23 | 397.18 | 39.00 | 0.45 | 0.05 | 16.00 | 12.00 | 1.00 | 1.00 | 2.00 | 8.33 | 16.68 | 60.00 | 2.00 | 12.00 | 1.00 |
| Liang(2022)b | 0.95 | 0.10 | 39.00 | 0.95 | 0.08 | 39.00 | 0.00 | 0.05 | 17.00 | 12.00 | 1.00 | 1.00 | 2.00 | 8.33 | 16.68 | 60.00 | 2.00 | 12.00 | 1.00 |
| Liu(2025)a | -427.91 | 66.85 | 36.00 | -527.27 | 343.62 | 33.00 | 0.41 | 0.06 | 18.00 | 13.00 | 1.00 | 0.00 | 2.00 | 14.74 | 20.91 | 60.00 | 2.00 | 12.00 | 1.00 |
| Liu(2025)b | 0.93 | 0.19 | 36.00 | 0.95 | 0.11 | 33.00 | -0.13 | 0.06 | 19.00 | 13.00 | 1.00 | 0.00 | 2.00 | 14.74 | 20.91 | 60.00 | 2.00 | 12.00 | 1.00 |
| Memarmoghaddam(2016)a | -1.21 | 1.03 | 19.00 | -1.29 | 0.84 | 17.00 | 0.08 | 0.11 | 20.00 | 14.00 | 1.00 | 1.00 | 1.00 | 8.30 | 16.72 | 90.00 | 3.00 | 8.00 | 1.00 |
| Memarmoghaddam(2016)b | -1168.89 | 92.76 | 19.00 | -1410.17 | 97.80 | 17.00 | 2.48 | 0.21 | 21.00 | 14.00 | 1.00 | 1.00 | 1.00 | 8.30 | 16.72 | 90.00 | 3.00 | 8.00 | 1.00 |
| Memarmoghaddam(2016)c | -352.78 | 30.33 | 19.00 | -363.41 | 30.86 | 17.00 | 0.34 | 0.11 | 22.00 | 14.00 | 1.00 | 1.00 | 1.00 | 8.30 | 16.72 | 90.00 | 3.00 | 8.00 | 1.00 |
| Memarmoghaddam(2016)d | -5.31 | 2.53 | 19.00 | -7.88 | 3.14 | 17.00 | 0.89 | 0.12 | 23.00 | 14.00 | 1.00 | 1.00 | 1.00 | 8.30 | 16.72 | 90.00 | 3.00 | 8.00 | 1.00 |
| Smith(2017)a | -125.00 | 129.00 | 42.00 | -160.00 | 108.00 | 38.00 | 0.29 | 0.05 | 24.00 | 15.00 |  | 1.00 | 2.00 | 7.41 |  | 120.00 | 3.00 | 15.00 | 2.00 |
| Smith(2017)b | -311.00 | 182.00 | 42.00 | -312.00 | 115.00 | 38.00 | 0.01 | 0.05 | 25.00 | 15.00 |  | 1.00 | 2.00 | 7.41 |  | 120.00 | 3.00 | 15.00 | 2.00 |
| Smith(2019) | 0.78 | 0.17 | 13.00 | 0.77 | 0.11 | 16.00 | 0.07 | 0.14 | 26.00 | 16.00 |  | 1.00 | 2.00 | 7.14 |  | 120.00 | 3.00 | 15.00 | 2.00 |

**Supplementary Table 6** Cognitive flexibility dataset

| study | mean1 | sd1 | n1 | mean2 | sd2 | n2 | es | se2 | aa | bb | inten | fre | type | age | bmi | duration | dura | week | wek |
| --- | --- | --- | --- | --- | --- | --- | --- | --- | --- | --- | --- | --- | --- | --- | --- | --- | --- | --- | --- |
| Barudin-Carreiro(2024)a | -56.86 | 11.67 | 7.00 | -56.50 | 10.28 | 8.00 | -0.03 | 0.27 | 1.00 | 1.00 |  | 0.00 | 0.00 | 9.67 |  | 20.00 | 1.00 | 1.00 | 1.00 |
| Barudin-Carreiro(2024)b | -56.71 | 12.66 | 7.00 | -56.20 | 10.70 | 8.00 | -0.04 | 0.27 | 2.00 | 1.00 |  | 0.00 | 0.00 | 9.67 |  | 20.00 | 1.00 | 1.00 | 1.00 |
| Benzing(2018)a | -324.00 | 177.00 | 24.00 | -495.00 | 214.00 | 22.00 | 0.86 | 0.10 | 3.00 | 2.00 | 1.00 | 0.00 | 2.00 | 10.48 | 18.46 | 15.00 | 1.00 | 1.00 | 1.00 |
| Benzing(2018)b | -909.00 | 200.00 | 24.00 | -1066.00 | 327.00 | 22.00 | 0.58 | 0.09 | 4.00 | 2.00 | 1.00 | 0.00 | 2.00 | 10.48 | 18.46 | 15.00 | 1.00 | 1.00 | 1.00 |
| Benzing and Schmidt(2019) | -874.00 | 156.00 | 28.00 | -1002.00 | 290.00 | 23.00 | 0.56 | 0.08 | 5.00 | 3.00 | 1.00 | 1.00 | 2.00 | 10.43 | 17.87 | 30.00 | 1.00 | 8.00 | 2.00 |
| Chang(2012)a | 4.70 | 2.07 | 20.00 | 4.85 | 1.92 | 20.00 | -0.07 | 0.10 | 6.00 | 4.00 | 0.00 | 0.00 | 0.00 | 10.43 | 16.79 | 30.00 | 1.00 | 1.00 | 1.00 |
| Chang(2012)b | -16.95 | 12.31 | 20.00 | -13.45 | 7.04 | 20.00 | -0.34 | 0.10 | 7.00 | 4.00 | 0.00 | 0.00 | 0.00 | 10.43 | 16.79 | 30.00 | 1.00 | 1.00 | 1.00 |
| Da Silva(2020) | 109.00 | 10.00 | 10.00 | 69.60 | 9.00 | 10.00 | 3.97 | 0.69 | 8.00 | 5.00 | 0.00 | 0.00 | 0.00 | 12.00 | 19.72 | 45.00 | 2.00 | 8.00 | 2.00 |
| Gerber(2012)a | -27.71 | 8.37 | 18.00 | -49.72 | 17.88 | 19.00 | 1.53 | 0.14 | 9.00 | 6.00 |  | 2.00 | 2.00 | 11.27 |  | 60.00 | 2.00 | 2.50 | 2.00 |
| Gerber(2012)b | -230.78 | 92.28 | 18.00 | -550.05 | 270.42 | 19.00 | 1.53 | 0.14 | 10.00 | 6.00 |  | 2.00 | 2.00 | 11.27 |  | 60.00 | 2.00 | 2.50 | 2.00 |
| Hattabi(2019)a | -21.50 | 12.89 | 20.00 | -38.25 | 12.89 | 20.00 | 1.27 | 0.12 | 11.00 | 7.00 | 0.00 | 1.00 | 0.00 | 9.85 | 17.55 | 90.00 | 3.00 | 12.00 | 2.00 |
| Hattabi(2019)b | -53.61 | 18.03 | 20.00 | -77.03 | 17.90 | 20.00 | 1.28 | 0.12 | 12.00 | 7.00 | 0.00 | 1.00 | 0.00 | 9.85 | 17.55 | 90.00 | 3.00 | 12.00 | 2.00 |
| Jun(2023)a | 8.24 | 1.77 | 26.00 | 8.24 | 1.44 | 24.00 | 0.00 | 0.08 | 13.00 | 8.00 | 1.00 | 2.00 | 1.00 |  |  | 40.00 | 2.00 | 6.00 | 2.00 |
| Jun(2023)b | 8.02 | 1.62 | 24.00 | 8.24 | 1.44 | 24.00 | -0.14 | 0.08 | 14.00 | 8.00 | 1.00 | 2.00 | 2.00 |  |  | 40.00 | 2.00 | 6.00 | 2.00 |
| Liang(2022)a | -177836.90 | 84188.13 | 39.00 | -247062.08 | 132891.93 | 39.00 | 0.62 | 0.05 | 15.00 | 9.00 | 1.00 | 1.00 | 2.00 | 8.33 | 16.68 | 60.00 | 2.00 | 12.00 | 2.00 |
| Liang(2022)b | -4.05 | 4.08 | 39.00 | -6.10 | 5.91 | 39.00 | 0.40 | 0.05 | 16.00 | 9.00 | 1.00 | 1.00 | 2.00 | 8.33 | 16.68 | 60.00 | 2.00 | 12.00 | 2.00 |
| Smith(2017)a | -0.34 | 0.13 | 42.00 | -0.30 | 0.08 | 38.00 | -0.36 | 0.05 | 17.00 | 10.00 |  | 1.00 | 2.00 | 7.41 |  | 120.00 | 3.00 | 15.00 | 2.00 |
| Smith(2017)b | -311.00 | 182.00 | 42.00 | -312.00 | 115.00 | 38.00 | 0.01 | 0.05 | 18.00 | 10.00 |  | 1.00 | 2.00 | 7.41 |  | 120.00 | 3.00 | 15.00 | 2.00 |

**Supplementary Table 7** Working memory dataset

| study | mean1 | sd1 | n1 | mean2 | sd2 | n2 | es | se2 | aa | bb | se | inten | fre | type | age | bmi | duration | dura | week | wek |
| --- | --- | --- | --- | --- | --- | --- | --- | --- | --- | --- | --- | --- | --- | --- | --- | --- | --- | --- | --- | --- |
| Benzing(2018) | 14.00 | 3.84 | 24.00 | 14.00 | 3.71 | 22.00 | 0.00 | 0.09 | 1.00 | 1.00 | 0.30 | 1.00 | 0.00 | 2.00 | 10.48 | 18.46 | 15.00 | 1.00 | 1.00 | 1.00 |
| Benzing and Schmidt(2019) | 15.54 | 3.80 | 28.00 | 14.44 | 3.92 | 23.00 | 0.28 | 0.08 | 2.00 | 2.00 | 0.28 | 1.00 | 1.00 | 2.00 | 10.43 | 17.87 | 30.00 | 1.00 | 8.00 | 2.00 |
| Bustamante(2016)a | 93.00 | 8.10 | 18.00 | 92.00 | 14.00 | 16.00 | 0.09 | 0.12 | 3.00 | 3.00 | 0.34 | 0.00 | 2.00 | 2.00 | 9.08 | 22.15 | 60.00 | 2.00 | 10.00 | 2.00 |
| Bustamante(2016)b | 95.00 | 17.00 | 18.00 | 96.00 | 15.00 | 16.00 | -0.06 | 0.12 | 4.00 | 3.00 | 0.34 | 0.00 | 2.00 | 2.00 | 9.08 | 22.15 | 60.00 | 2.00 | 10.00 | 2.00 |
| Hattabi(2019)a | -12.65 | 2.13 | 20.00 | -18.70 | 2.72 | 20.00 | 2.43 | 0.18 | 5.00 | 4.00 | 0.43 | 0.00 | 1.00 | 0.00 | 9.85 | 17.55 | 90.00 | 3.00 | 12.00 | 2.00 |
| Hattabi(2019)b | -87.55 | 36.30 | 20.00 | -105.15 | 28.37 | 20.00 | 0.53 | 0.10 | 6.00 | 4.00 | 0.32 | 0.00 | 1.00 | 0.00 | 9.85 | 17.55 | 90.00 | 3.00 | 12.00 | 2.00 |
| Huang(2024)a | 76.99 | 14.61 | 22.00 | 76.84 | 15.90 | 17.00 | 0.01 | 0.10 | 7.00 | 5.00 | 0.32 | 1.00 | 0.00 | 0.00 | 9.64 | 18.58 | 60.00 | 2.00 | 8.00 | 2.00 |
| Huang(2024)b | -1901.82 | 857.05 | 22.00 | -1886.91 | 695.83 | 17.00 | -0.02 | 0.10 | 8.00 | 5.00 | 0.32 | 1.00 | 0.00 | 0.00 | 9.64 | 18.58 | 60.00 | 2.00 | 8.00 | 2.00 |
| Huang(2024)c | 69.53 | 23.37 | 16.00 | 76.84 | 15.90 | 17.00 | -0.36 | 0.12 | 9.00 | 5.00 | 0.35 | 1.00 | 0.00 | 2.00 | 9.15 | 18.03 | 60.00 | 2.00 | 8.00 | 2.00 |
| Huang(2024)d | -1905.45 | 950.15 | 16.00 | -1886.91 | 695.83 | 17.00 | -0.02 | 0.12 | 10.00 | 5.00 | 0.35 | 1.00 | 0.00 | 2.00 | 9.15 | 18.03 | 60.00 | 2.00 | 8.00 | 2.00 |
| Huang(2025) | -23.33 | 13.58 | 12.00 | -41.50 | 21.99 | 12.00 | 0.96 | 0.19 | 11.00 | 6.00 | 0.44 | 1.00 | 0.00 | 1.00 | 10.21 | 18.04 | 80.00 | 3.00 | 24.00 | 2.00 |
| Jun(2023)a | 3.15 | 0.26 | 26.00 | 3.15 | 8.56 | 24.00 | 0.00 | 0.08 | 12.00 | 7.00 | 0.28 | 1.00 | 2.00 | 1.00 |  |  | 40.00 | 2.00 | 6.00 | 2.00 |
| Jun(2023)b | 3.82 | 0.33 | 24.00 | 3.15 | 8.56 | 24.00 | 0.11 | 0.08 | 13.00 | 7.00 | 0.29 | 1.00 | 2.00 | 2.00 |  |  | 40.00 | 2.00 | 6.00 | 2.00 |
| Liang(2022)a | -22986.59 | 7924.86 | 39.00 | -27104.65 | 9221.73 | 39.00 | 0.47 | 0.05 | 14.00 | 8.00 | 0.23 | 1.00 | 1.00 | 2.00 | 8.33 | 16.68 | 60.00 | 2.00 | 12.00 | 2.00 |
| Liang(2022)b | 29.54 | 3.01 | 39.00 | 26.51 | 3.40 | 39.00 | 0.93 | 0.06 | 15.00 | 8.00 | 0.24 | 1.00 | 1.00 | 2.00 | 8.33 | 16.68 | 60.00 | 2.00 | 12.00 | 2.00 |
| Ludyga(2022)a | -1057.40 | 264.50 | 29.00 | -954.10 | 261.20 | 28.00 | -0.39 | 0.07 | 16.00 | 9.00 | 0.27 | 1.00 | 0.00 | 1.00 | 10.39 | 18.25 | 60.00 | 2.00 | 12.00 | 2.00 |
| Ludyga(2022)b | 2.00 | 0.50 | 29.00 | 1.90 | 0.90 | 28.00 | 0.14 | 0.07 | 17.00 | 9.00 | 0.27 | 1.00 | 0.00 | 1.00 | 10.39 | 18.25 | 60.00 | 2.00 | 12.00 | 2.00 |
| Smith(2017)a | 12.30 | 3.20 | 42.00 | 11.80 | 3.10 | 38.00 | 0.16 | 0.05 | 18.00 | 10.00 | 0.22 |  | 1.00 | 2.00 | 7.41 |  | 120.00 | 3.00 | 15.00 | 2.00 |
| Smith(2017)b | 10.50 | 3.00 | 42.00 | 10.40 | 3.60 | 38.00 | 0.03 | 0.05 | 19.00 | 10.00 | 0.22 |  | 1.00 | 2.00 | 7.41 |  | 120.00 | 3.00 | 15.00 | 2.00 |

**Supplementary Table 8** Internalizing symptoms dataset

| study | mean1 | sd1 | n1 | mean2 | sd2 | n2 | es | se2 | aa | bb | inten | fre | type | age | bmi | duration | dura | week | wek |
| --- | --- | --- | --- | --- | --- | --- | --- | --- | --- | --- | --- | --- | --- | --- | --- | --- | --- | --- | --- |
| Da Silva(2020)a | -15.70 | 3.00 | 10.00 | -13.30 | 4.00 | 10.00 | -0.65 | 0.21 | 1.00 | 1.00 | 0.00 | 0.00 | 0.00 | 12.00 | 19.72 | 45.00 | 2.00 | 8.00 | 2.00 |
| Da Silva(2020)b | -10.20 | 2.00 | 10.00 | -16.40 | 3.00 | 10.00 | 2.33 | 0.37 | 2.00 | 1.00 | 0.00 | 0.00 | 0.00 | 12.00 | 19.72 | 45.00 | 2.00 | 8.00 | 2.00 |
| Da Silva(2020)c | -23.00 | 2.00 | 10.00 | -32.00 | 3.00 | 10.00 | 3.38 | 0.56 | 3.00 | 1.00 | 0.00 | 0.00 | 0.00 | 12.00 | 19.72 | 45.00 | 2.00 | 8.00 | 2.00 |
| Hattabi(2022)a | -12.55 | 2.04 | 20.00 | -20.00 | 7.14 | 20.00 | 1.39 | 0.13 | 4.00 | 2.00 | 0.00 | 1.00 | 0.00 | 9.85 | 17.55 | 90.00 | 3.00 | 12.00 | 2.00 |
| Hattabi(2022)b | -4.50 | 0.95 | 20.00 | -7.60 | 2.89 | 20.00 | 1.41 | 0.13 | 5.00 | 2.00 | 0.00 | 1.00 | 0.00 | 9.85 | 17.55 | 90.00 | 3.00 | 12.00 | 2.00 |
| Hattabi(2022)c | -4.50 | 0.95 | 20.00 | -7.60 | 2.89 | 20.00 | 1.41 | 0.13 | 6.00 | 2.00 | 0.00 | 1.00 | 0.00 | 9.85 | 17.55 | 90.00 | 3.00 | 12.00 | 2.00 |
| Hattabi(2022)d | -4.20 | 0.83 | 20.00 | -6.65 | 2.50 | 20.00 | 1.29 | 0.12 | 7.00 | 2.00 | 0.00 | 1.00 | 0.00 | 9.85 | 17.55 | 90.00 | 3.00 | 12.00 | 2.00 |
| Hattabi(2022)e | -3.85 | 0.59 | 20.00 | -5.75 | 2.00 | 20.00 | 1.26 | 0.12 | 8.00 | 2.00 | 0.00 | 1.00 | 0.00 | 9.85 | 17.55 | 90.00 | 3.00 | 12.00 | 2.00 |
| Hoza(2015)a | -0.90 | 0.76 | 49.00 | -0.83 | 0.74 | 45.00 | -0.09 | 0.04 | 9.00 | 3.00 | 1.00 | 2.00 | 2.00 | 6.83 | 17.29 | 31.00 | 2.00 | 12.00 | 2.00 |
| Hoza(2015)b | -0.40 | 0.65 | 49.00 | -0.27 | 0.48 | 45.00 | -0.22 | 0.04 | 10.00 | 3.00 | 1.00 | 2.00 | 2.00 | 6.83 | 17.29 | 31.00 | 2.00 | 12.00 | 2.00 |
| Jovicevic(2024) | -15.43 | 2.56 | 20.00 | -16.35 | 2.59 | 19.00 | 0.35 | 0.10 | 11.00 | 4.00 |  | 0.00 | 1.00 | 10.00 |  | 60.00 | 2.00 | 12.00 | 2.00 |
| Liang(2024) | -5.53 | 4.45 | 15.00 | -10.40 | 7.71 | 15.00 | 0.75 | 0.14 | 12.00 | 5.00 | 1.00 | 1.00 | 1.00 | 8.62 | 16.30 | 60.00 | 2.00 | 12.00 | 2.00 |
| Liu(2025)a | -3.35 | 2.95 | 36.00 | -5.65 | 4.37 | 33.00 | 0.62 | 0.06 | 13.00 | 6.00 | 1.00 | 0.00 | 2.00 | 14.74 | 20.91 | 60.00 | 2.00 | 12.00 | 2.00 |
| Liu(2025)b | -4.35 | 2.67 | 36.00 | -6.08 | 4.17 | 33.00 | 0.49 | 0.06 | 14.00 | 6.00 | 1.00 | 0.00 | 2.00 | 14.74 | 20.91 | 60.00 | 2.00 | 12.00 | 2.00 |
| Liu(2025)c | -3.38 | 2.79 | 36.00 | -6.18 | 4.34 | 33.00 | 0.77 | 0.06 | 15.00 | 6.00 | 1.00 | 0.00 | 2.00 | 14.74 | 20.91 | 60.00 | 2.00 | 12.00 | 2.00 |
| Oh(2018)a | -57.63 | 10.45 | 17.00 | -63.86 | 12.61 | 15.00 | 0.53 | 0.13 | 16.00 | 7.00 | 0.00 | 0.00 | 1.00 | 8.15 |  | 60.00 | 2.00 | 12.00 | 2.00 |
| Oh(2018)b | -59.69 | 7.95 | 17.00 | -65.14 | 10.81 | 15.00 | 0.57 | 0.13 | 17.00 | 7.00 | 0.00 | 0.00 | 1.00 | 8.15 |  | 60.00 | 2.00 | 12.00 | 2.00 |
| Oh(2018)c | -57.75 | 7.73 | 17.00 | -64.07 | 13.38 | 15.00 | 0.57 | 0.13 | 18.00 | 7.00 | 0.00 | 0.00 | 1.00 | 8.15 |  | 60.00 | 2.00 | 12.00 | 2.00 |
| Oh(2018)d | -59.69 | 7.95 | 17.00 | -65.14 | 10.81 | 15.00 | 0.57 | 0.13 | 19.00 | 7.00 | 0.00 | 0.00 | 1.00 | 8.15 |  | 60.00 | 2.00 | 12.00 | 2.00 |
| Oh(2018)e | -57.00 | 9.35 | 17.00 | -60.14 | 8.56 | 15.00 | 0.34 | 0.13 | 20.00 | 7.00 | 0.00 | 0.00 | 1.00 | 8.15 |  | 60.00 | 2.00 | 12.00 | 2.00 |
| Salleg-Cabarcas(2024)a | 3.30 | 0.63 | 22.00 | 0.46 | 0.84 | 22.00 | 3.74 | 0.27 | 21.00 | 8.00 | 1.00 | 1.00 | 1.00 | 10.50 |  | 45.00 | 2.00 | 12.00 | 2.00 |
| Salleg-Cabarcas(2024)b | 1.13 | 0.34 | 22.00 | 0.08 | 0.46 | 22.00 | 2.57 | 0.17 | 22.00 | 8.00 | 1.00 | 1.00 | 1.00 | 10.50 |  | 45.00 | 2.00 | 12.00 | 2.00 |

**Supplementary Table 9** Externalizing symptoms dataset

| study | mean1 | sd1 | n1 | mean2 | sd2 | n2 | es | se2 | aa | bb |
| --- | --- | --- | --- | --- | --- | --- | --- | --- | --- | --- |
| Bustamante(2016)a | -103.00 | 20.00 | 14.00 | -104.00 | 12.00 | 13.00 | 0.06 | 0.15 | 1.00 | 1.00 |
| Bustamante(2016)b | -0.90 | 0.70 | 14.00 | -0.60 | 0.20 | 13.00 | -0.56 | 0.16 | 2.00 | 1.00 |
| Hattabi(2022)a | -14.65 | 2.85 | 20.00 | -24.70 | 1.38 | 20.00 | 4.40 | 0.37 | 3.00 | 2.00 |
| Hattabi(2022)b | -9.75 | 1.45 | 20.00 | -16.35 | 1.09 | 20.00 | 5.04 | 0.45 | 4.00 | 2.00 |
| Hattabi(2022)c | -4.90 | 1.65 | 20.00 | -8.35 | 0.81 | 20.00 | 2.60 | 0.19 | 5.00 | 2.00 |
| Hoza(2015)a | -1.27 | 0.68 | 49.00 | -1.07 | 0.57 | 45.00 | -0.31 | 0.04 | 6.00 | 3.00 |
| Hoza(2015)b | -0.63 | 0.67 | 49.00 | -0.57 | 0.71 | 45.00 | -0.09 | 0.04 | 7.00 | 3.00 |
| Hoza(2015)c | 0.76 | 0.56 | 49.00 | 0.69 | 0.52 | 45.00 | 0.13 | 0.04 | 8.00 | 3.00 |
| Hoza(2015)d | 0.62 | 0.60 | 49.00 | 0.58 | 0.57 | 45.00 | 0.07 | 0.04 | 9.00 | 3.00 |
| Jovicevic(2024) | -15.43 | 2.56 | 20.00 | -16.35 | 2.59 | 19.00 | 0.35 | 0.10 | 10.00 | 4.00 |
| Liang(2024) | -10.73 | 5.75 | 15.00 | -14.93 | 6.83 | 15.00 | 0.65 | 0.14 | 11.00 | 5.00 |
| Liu(2025) | -22.12 | 9.76 | 36.00 | -26.15 | 10.95 | 33.00 | 0.39 | 0.06 | 12.00 | 6.00 |
| Oh(2018)a | -60.94 | 11.95 | 17.00 | -64.29 | 11.97 | 15.00 | 0.27 | 0.13 | 13.00 | 7.00 |
| Oh(2018)b | -61.63 | 10.07 | 17.00 | -62.86 | 11.48 | 15.00 | 0.11 | 0.13 | 14.00 | 7.00 |
| Oh(2018)c | -57.06 | 6.99 | 17.00 | -62.71 | 7.05 | 15.00 | 0.78 | 0.14 | 15.00 | 7.00 |
| Salleg-Cabarcas(2024)a | 2.09 | 0.76 | 22.00 | 0.46 | 1.02 | 22.00 | 1.78 | 0.13 | 16.00 | 8.00 |
| Salleg-Cabarcas(2024)b | -0.54 | 1.14 | 22.00 | 2.52 | 0.86 | 22.00 | -2.98 | 0.20 | 17.00 | 8.00 |

Taking the cognitive flexibility outcome as an example, the R code for the main effects analysis is as follows:
